# Three New Isoflavonoid Glycosides from the Mangrove-Derived Actinomycete *Micromonospora aurantiaca* 110B

**DOI:** 10.3390/md17050294

**Published:** 2019-05-17

**Authors:** Rui-Jun Wang, Shao-Yong Zhang, Yang-Hui Ye, Zhen Yu, Huan Qi, Hui Zhang, Zheng-Lian Xue, Ji-Dong Wang, Min Wu

**Affiliations:** 1 Ocean College, Zhejiang University, Zhoushan 316021, China; 15034663445@163.com (R.-J.W.); yyhleslie@163.com (Y.-H.Y.); 2 College of Biochemical Engineering, Anhui Polytechnic University, Wuhu 241000, China; xuezhen0851@sina.com; 3 Zhejiang Key Laboratory of Antifungal Drugs, Zhejiang Hisun Pharmaceutical Co., Ltd., Taizhou 318000, China; 1zhangshaoyong@163.com (S.-Y.Z.); yuzhen@hisunpharm.com (Z.Y.); qihuan@hisunpharm.com (H.Q.); huizhang@hisunpharm.com (H.Z.)

**Keywords:** *Micromonospora aurantiaca* 110B, isoflavonoid glycosides, structure elucidation, cytotoxic activity

## Abstract

The mangrove ecosystem is a rich resource for the discovery of actinomycetes with potential applications in pharmaceutical science. Besides the genus *Streptomyces*, *Micromonospora* is also a source of new bioactive agents. We screened *Micromonospora* from the rhizosphere soil of mangrove plants in Fujian province, China, and 51 strains were obtained. Among them, the extracts of 12 isolates inhibited the growth of human lung carcinoma A549 cells. Strain 110B exhibited better cytotoxic activity, and its bioactive constituents were investigated. Consequently, three new isoflavonoid glycosides, daidzein-4′-(2-deoxy-*α*-l-fucopyranoside) (**1**), daidzein-7-(2-deoxy-*α*-l-fucopyranoside) (**2**), and daidzein-4′,7-di-(2-deoxy-*α*-l-fucopyranoside) (**3**) were isolated from the fermentation broth of strain 110B. The structures of the new compounds were determined by spectroscopic methods, including 1D and 2D nuclear magnetic resonance (NMR) and high-resolution electrospray ionization mass spectrometry (HR-ESIMS). The result of medium-changing experiments implicated that these new compounds were microbial biotransformation products of strain *M. aurantiaca* 110B. The three compounds displayed moderate cytotoxic activity to the human lung carcinoma cell line A549, hepatocellular liver carcinoma cell line HepG2, and the human colon tumor cell line HCT116, whereas none of them showed antifungal or antibacterial activities.

## 1. Introduction

The mangrove ecosystem, distinguished by its land-to-ocean transition, is complex and special. Mangroves thrive in a stressful environment of high salinity, high moisture, strong winds, and high osmotic pressure [1]. This environment breeds a distinctive microbial community that can tolerate numerous stresses and has developed unique metabolic pathways for the purpose of structural ecological adaptability [2,3]. Recent research has focused on the diversity of mangrove microorganisms and their metabolic compounds [4,5,6]. Among mangrove microorganisms, actinomycetes have gained more attention due to their ability to produce impressive numbers of bioactive metabolites [3]. There is clear evidence that the mangrove ecosystem contains a large diversity of actinomycetes [7]. As one of the genera of actinomycetes, the genus *Micromonospora* is an important prolific producer of secondary metabolites [8,9].

The genus *Micromonospora*, belonging to the family *Micromonosporaceae*, was proposed by Ørskov [10]. Currently, this genus consists of more than 80 recognized species, according to the List of Prokaryotic Names with Standing in Nomenclature (LPSN) [11]. About 20 new species of *Micromonospora* have been isolated in the last five years. Many of them were isolated from mangrove environments, such as *Micromonospora sediminis* CH3-3^T^ [12], *Micromonospora ovatispora* 2701SIM06^T^ [13], *Micromonospora zhanjiangensis* 2902at01^T^ [14], *Micromonospora sonneratiae* 274745^T^ [15], and *Micromonospora maritima* D10-9-5^T^ [16]. Meanwhile, some new metabolites with biological activities have been isolated from the mangrove-derived *Micromonospora* strains. *Micromonospora rifamycinica* AM105 produced rifamycin S and the geometric isomer of rifamycin S with antimicrobial activity against methicillin-resistant *Staphylococcus aureus* (MRSA) [17]. Butremycin was isolated from *Micromonospora* sp. K310 and displayed weak antibacterial activity against Gram-positive *Staphylococcus aureus* ATCC 25923 and Gram-negative *Escherichia coli* ATCC 25922 [18]. 3-Methyl-carboline from *Micromonospora* sp. M2DG17 showed weak inhibitory activity on human colon carcinoma HCT 116 cell lines [19].

In our continuing study of mangrove actinomycetes, 51 strains of *Micromonospora* were isolated from the rhizosphere soil of mangrove plants in Fujian province. The crude extracts obtained from the isolates were evaluated for cytotoxic activity. Based on the cytotoxic activity, further chemical investigations were performed on *Micromonospora aurantiaca* 110B. As a result, three new isoflavonoid glycosides (**1**–**3**) were isolated from its fermentation broth. Herein, we describe the isolation, structure elucidation, and cytotoxic activity of these three new compounds.

## 2. Results

### 2.1. Isolation and Screening of Strains for Cytotoxic Activities

**Figure 1:**
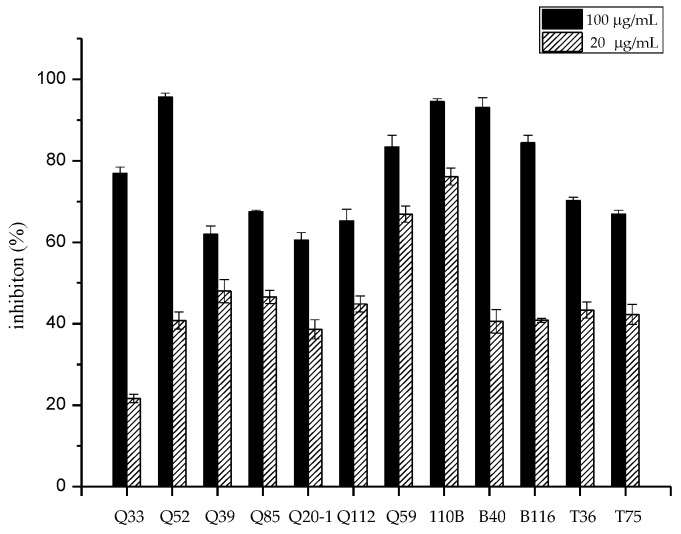
Cytotoxic activity of extracts obtained from 12 isolates against the human lung tumor cell line A549 in vitro.

**Figure 2:**
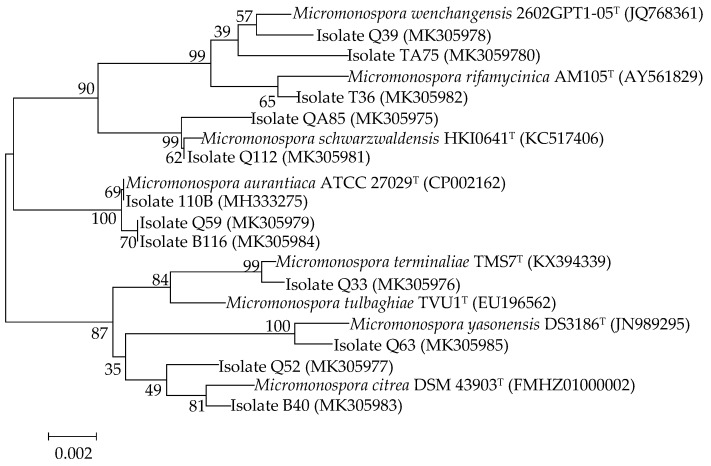
Neighbor-joining phylogenetic tree based on nearly complete 16S rRNA gene sequences showing relationships between the 12 active isolates and the type strains of the highest 16S rDNA sequence similarity. Bootstrap values were based on 1000 replicates; only values ≥50% are shown. Bar: 0.002 substitutions per nucleotide position.

Fifty-one strains belonging to the genus *Micromonospora* were isolated from mangrove soil samples. The crude extracts of these isolates were examined for their cytotoxic activity. After primary screening, 12 strains showed cytotoxic activity to the human lung tumor cell line A549 (Figure 1). The 16S rRNA genes of the 12 strains were sequenced and the sequences were blasted against GenBank (https://www.ncbi.nlm.nih.gov/genbank/). The results showed that the isolates belonged to seven different species (Figure 2) and that strain 110B was closely related to the type strain *Micromonospora aurantiaca* ATCC 27029^T^ (CP002162).

Due to the superior cytotoxic activity of strain 110B, further chemical investigations were performed on this strain.

### 2.2. Structural Elucidation

The strain 110B was grown in a preparative scale in 30 L of fermentation medium for 7 days. A chemical investigation of its fermentation broth led to the isolation of three new isoflavonoid glycosides (compounds **1**–**3**).

**Figure 3:**
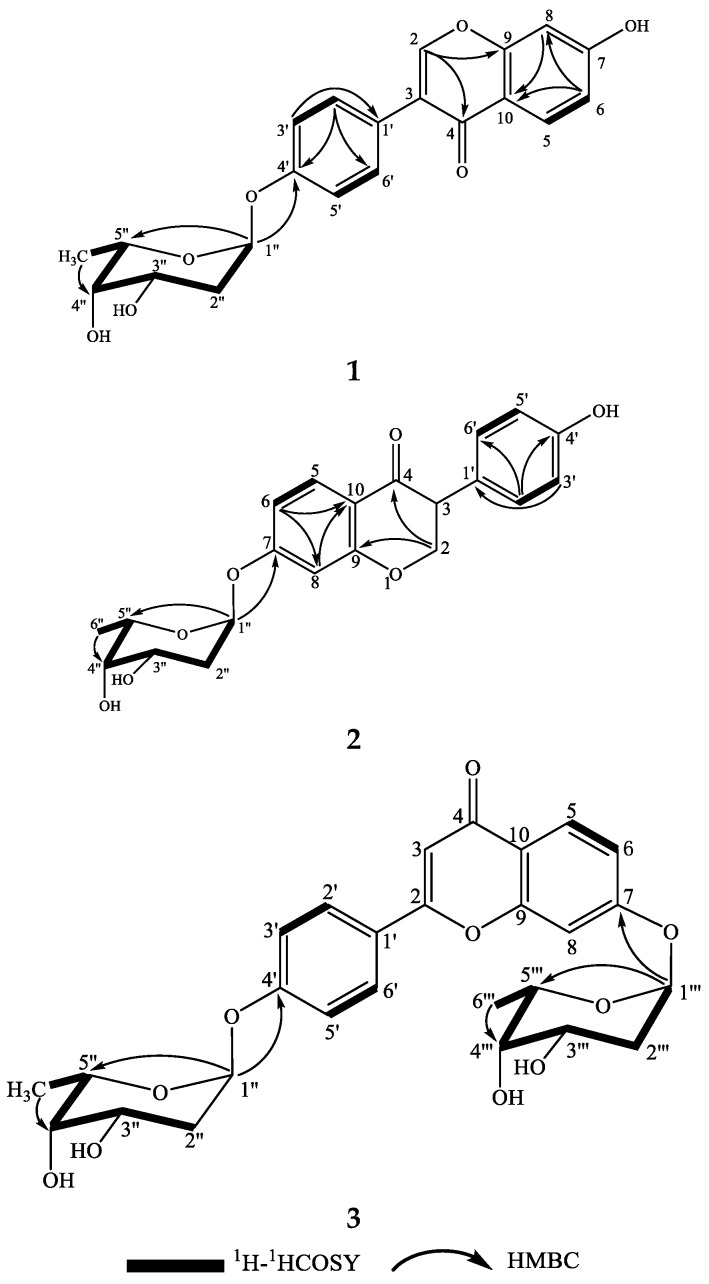
The structures and key ^1^H–^1^H COSY and HMBC correlations for compounds **1**–**3**.

**Figure 4:**
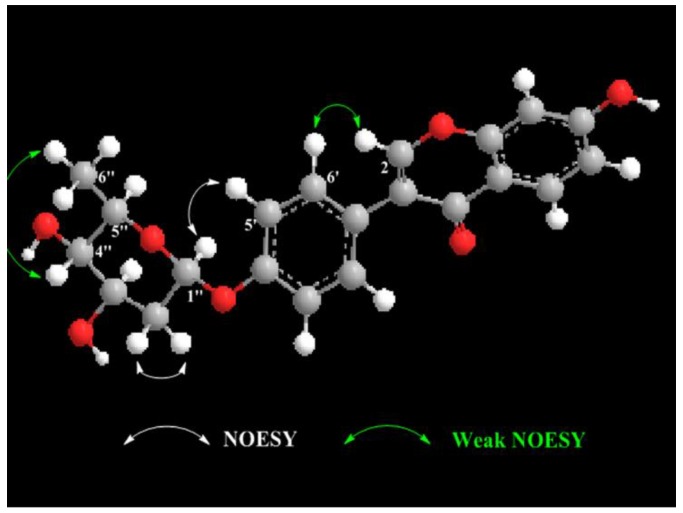
The key NOESY correlations for compound **1**.

Compound **1** was obtained as a white powder with a negative specific rotation value ([*α*${]}_{D}^{25}$−107, EtOH) and UV (EtOH) *λ*_max_ (log *ε*) 249 (4.49), 301 (4.10) nm. The molecular formula of **1** was established to be C_21_H_20_O_7_ from positive-ion HR-ESIMS (Figure S1) and ^13^C NMR spectral (Figure S4) data (Table 1). The IR spectrum (Figure S2) of **1** revealed carbonyl group absorption at 1628 cm^−1^ and hydroxy group absorption at 3353 cm^−1^. The ^1^H NMR (Figure S3) data (Table 1) showed the characteristic isoflavone signal for H-2 at *δ*_H_ 8.08 (1H, s); two sets of aromatic hydrogen peaks at *δ*_H_ 7.39 (2H, d, *J* = 8.7 Hz) and *δ*_H_ 7.05 (2H, d, *J* = 8.7 Hz) corresponding to a 4-hydroxyphenyl moiety (B ring); three aromatic protons corresponding to a 7-substituted A ring at *δ*_H_ 7.97 (1H, d, *J* = 9.0 Hz), 6.84 (1H, dd, *J* = 9.0, 2.4 Hz), and *δ*_H_ 6.76 (1H, d, *J* = 2.4 Hz); and nine proton signals attributable to sugars at *δ*_H_ 5.62 (1H, br d, *J* = 3.5 Hz), 4.09 (1H, ddd, *J* = 11.9, 5.0, 3.0 Hz), 3.89 (1H, q, *J* = 6.6 Hz), 3.55 (1H, br d, *J* = 3.0 Hz), 2.03 (1H, ddd, *J* = 12.8, 11.9, 3.6 Hz), 1.90 (1H, dd *J* = 12.8, 5.0 Hz), and *δ*_H_ 1.10 (3H, d, *J* = 6.6 Hz). The ^13^C NMR spectrum (Table 1) displayed 21 carbon resonances, including 15 carbons for the isoflavone skeleton and 6 carbons for the sugar. The NMR spectral (Figure S6) data suggested that compound **1** was an isoflavone glycoside, and the isoflavone skeleton proved to be daidzein based on a comparison of the spectroscopic data with previously reported values [20,21]. The 6-carbon resonances of sugar can be categorized as 1 methyl (*δ*_C_ 18.4), 1 methylene (*δ*_C_ 32.09), 1 acetal (*δ*_C_ 97.6), and 3 methines (*δ*_C_ 72.1, 68.6, 66.7) by ^13^C NMR and distortionless enhancement by polarization transfer (DEPT, Figure S5) experiments, indicating the presence of a 2,6-dideoxysugar moiety. The correlations of H-1″/H_2_-2″/H-3″/H-4″/H-5″/H_3_-6″ in the ^1^H–^1^H correlation spectroscopy (COSY) spectrum (Figure 3 and Figure S7) and the observed heteronuclear multiple bond correlation (HMBC) signals (Figure 3 and Figure S8) from H-1″ to C-5″ and H_3_-6″ to C-4″ further established the 2,6-dideoxysugar structure. In the HMBC experiment, the correlation between the anomeric proton (H-1″) and the carbon at *δ*_C_ 158.3 (C-4′) confirmed the attachment of the sugar moiety to the phenolic 4′-*O*-atom. From the detailed analysis of the ^1^H NMR spectrum, the small coupling constant of H-1″ (*δ*_H_ 5.62 br d, *J* = 3.5 Hz) revealed that the sugar is *α*-glycosidically linked to the phenolic oxygen atom [22]. The signal patterns of H-3″ (*δ*_H_ 4.09 ddd, *J* = 11.9, 5.0, 3.0 Hz) and H-4″ (*δ*_H_ 3.55 br d, *J* = 3.0 Hz) showed equatorial 3″-OH and axial 4″-OH groups. Further, signals at H-4″ exhibited 3.0 coupling, which could be assigned to the axial H-5″ and axial H-3″, respectively. This 2,6-dideoxysugar was assumed to be 2-deoxy-l-fucose on the basis of ^1^H and ^13^C NMR data [23,24] and the NOESY experiment (Figure S9). Acid hydrolysis of compound **1** afforded the aglycone daidzein and a 2-deoxy-l-fucose. The aglycone fraction was identified by HPLC analysis. The retention time of the aglycone moiety of **1** was in good agreement with that of daidzein (Figure S28). The sugar fraction liberated by acid hydrolysis was treated with l-cysteine methyl ester, followed by reaction with *O*-tolylisothiocyanate. By reversed-phase HPLC analysis, the identical retention time of the liberated sugar derivative with that of the 2-deoxy-l-fucose derivative indicated that the sugar moiety of **1** was 2-deoxy-fucose (Figure S29). Consequently, the structure of **1** was elucidated to be daidzein-4′-(2-deoxy-*α*-l-fucopyranoside) (Figure 3 and Figure 4).

Compound **2** was obtained as a white powder with a negative specific rotation value ([*α*${]}_{D}^{25}$−112, EtOH). The molecular formula of **2** was established as C_21_H_20_O_7_ from positive-ion HR-ESIMS (Figure S10) and ^13^C NMR spectral data (Table 1). Bands for hydroxy group at 3357 cm^−1^ and the carbonyl group at 1634 cm^−1^ were evident in the IR spectrum (Figure S11). In the ^1^H NMR spectrum (Table 1) of **2**, the characteristic H-2 signal of an isoflavone was exhibited at *δ*_H_ 8.12 (1H, s), and the 4-hydroxyphenyl nature of the B ring was deduced from the signals at *δ*_H_ 7.31 (2H, d, *J* = 9.0 Hz) and *δ*_H_ 6.78 (2H, d, *J* = 9.0 Hz). Three aromatic proton signals were observed at *δ*_H_ 8.06 (1H, d, *J* = 9.0 Hz), 7.18 d (1H, d, *J* = 2.3 Hz), and *δ*_H_ 7.09 (1H, dd, *J* = 9.0, 2.3 Hz), indicating that the A ring was substituted at C-7. An anomeric proton signal at *δ*_H_ 5.78 d (1H, br d, *J* = 3.6 Hz) also appeared in the ^1^H NMR spectrum of **2**. Comparison of the NMR data (Figures S12–S18) of **2** with those of **1** revealed close similarities, except for the substituted pattern of the 2,6-dideoxysugar moiety. The observed HMBC correlation (Figure 3) from the anomeric proton H-1″ to C-7 (*δ*_C_ 163.1) confirmed the position of the 2,6-dideoxysugar. The 2,6-dideoxysugar was deduced to be 2-deoxy-l-fucose according to HPLC analysis of the corresponding *O*-tolylthiocarbamate derivative of the liberated sugar, as described above. Thus, the structure of **2** was identified as daidzein-7-(2-deoxy-*α*-l-fucopyranoside) (Figure 3).

Compound **3** was obtained as a white powder with a negative specific rotation value ([*α*${]}_{D}^{25}$−27.0, EtOH). The molecular formula of **3** was deduced to be C_27_H_30_O_10_ from positive-ion HR-ESIMS (Figure S19) and ^13^C NMR spectral data (Table 1). The IR spectrum (Figure S20) revealed carbonyl absorption at 1650 cm^−1^ and hydroxy absorption at 3381 cm^−1^. The ^1^H NMR spectrum of **3** (Table 1) showed eight aromatic proton signals at *δ*_H_ 8.21 (1H, s), 8.12 (1H, d, *J* = 8.9 Hz), 7.47 (2H, d, *J* = 8.7 Hz), 7.25 (2H, d, *J* = 2.2 Hz), 7.15 (1H, dd, *J* = 8.9, 2.2 Hz), and *δ*_H_ 7.13 (1H, d, *J* = 8.7 Hz), indicating that the isoflavone aglycone of **3** was identical to **1** and **2**. Two sugar moieties were evident from the anomeric proton signals at *δ*_H_ 5.84 (1H, br d, *J* = 3.5 Hz) and *δ*_H_ 5.69 (1H, br d, *J* = 3.5 Hz) in the ^1^H NMR spectrum. The ^1^H and ^13^C NMR spectral (Figures S21–S23) data of **3** were similar to those of **1** and **2**, except for an extra 2,6-dideoxysugar moiety. The HMBC (Figure S26) correlations from H-1″ to C-4′ and H-1‴ to C-7 revealed that the two 2,6-dideoxysugar moieties were located at C-4′ and C-7, respectively. The sugar moieties of **3** were determined by HPLC analysis of the *O*-tolylthiocarbamate derivative of the liberated sugar from acid hydrolysis and a standard 2-deoxy-l-fucose derivative. So, the structure of **3** was determined to be daidzein-4′,7-di-(2-deoxy-*α*-l-fucopyranoside), as shown in Figure 3.

**Figure 5:**
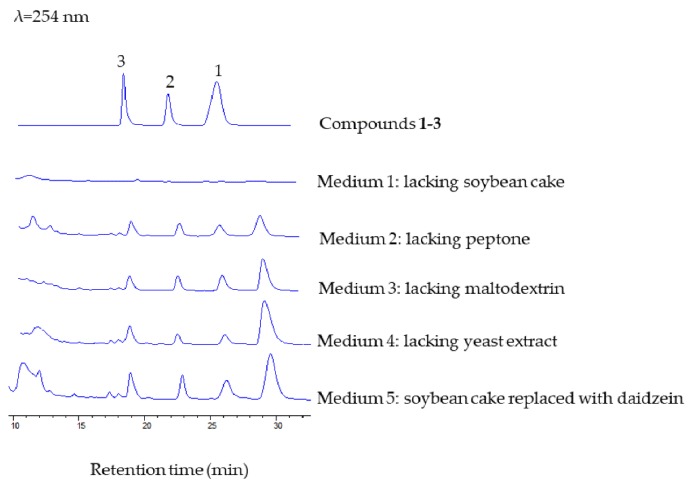
HPLC analysis of the formation of compounds **1**–**3** in different media, with a mobile phase of CH_3_CN/H_2_O (25:75, v/v), flow rate of 1.5 mL/min^−1^, and detection wavelength at 254 nm.

Isoflavonoids have been frequently isolated from bacterial and fungal cultures, but their biosynthetic origin has not been unveiled in all cases. Not all of the isoflavonoids were synthesized ‘de novo’. Ndejouong and Hertweck found that *Streptomyces mirabilis* could transform isoflavones into hydroxylated and reduced derivatives [25]. In order to clarify whether compounds **1**–**3** were synthesized ‘de novo’, we carried out a series of experiments. We prepared different media (media 1–4) to explore whether the ingredients in the fermentation medium will affect the production of compounds **1**–**3**, and we mainly focused on the medium that lacked soybean cake or peptone. We found that the three new compounds were only detected in medium containing soybean cake. Then, when daidzein was added to the medium lacking soybean cake (giving medium 5), we could observe formation of these new compounds (Figure 5). These results indicated that *M. aurantiaca* 110B could transform plant daidzein into fucosylated derivatives.

### 2.3. Analysis of Deoxy-Sugar Glycosyltransferases

**Figure 6:**
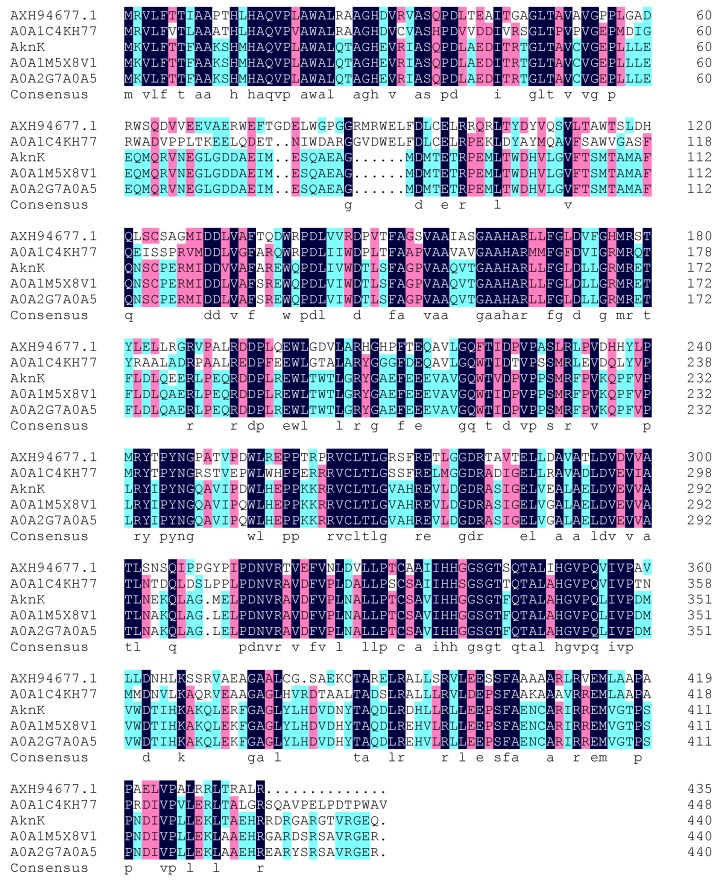
Alignment of the deduced amino acid sequence of AXH94677.1 in strain 110B with different 2-deoxyfucosyltransferases: A0A1C4KH77 (SCD60843.1) from *Streptomyces* sp. DvalAA-19, AknK (AAF70102.1) from *Streptomyces galilaeus*, A0A1M5X8V1 (SHH96196.1) from *Streptomyces* sp. 3214.6, and A0A2G7A0A5 (PIG12760.1) from *Streptomyces* sp. 1121.2.

The genome of the strain 110B was sequenced and analyzed. We focused on whether there were any deoxy-sugar-related enzymes in strain *M. aurantiaca* 110B because the sugar moieties of the three new isoflavonoid glycosides were 2,6-dideoxysugars. Deoxy-sugar glycosyltransferases are interesting enzymes because of their important role in many natural product drugs. Glycosyltransferase-related protein sequences of the *M. aurantiaca* 110B were aligned with protein sequences of the UniProt database [26,27], and one protein (AXH94677.1) similar to AknK was found (Figure 6). AknK is an l-2-deoxy-l-fucose transferase found in *Streptomyces galilaeus*, which catalyzes the addition of the second sugar to aclacinomycin A [28]. The genome information of strain 110B also showed that the sugar moieties of the three new isoflavonoid glycosides might be 2-deoxy-fucose. This conclusion was consistent with the results of the structural analysis.

### 2.4. Biological Activity

Compounds **1**–**3** were tested for their activity against the proliferation of the human hepatocellular liver carcinoma cell line HepG2, human lung tumor cell line A549, and human colon tumor cell line HCT116. The results (Table 2) showed that the three compounds exhibited moderate cytotoxic activity against the three cell lines.

The antifungal and antibacterial properties of the three compounds were tested against three human pathogens: the fungus *Candida albicans*, the Gram-positive bacterium methicillin-resistant *S. aureus*, and the Gram-negative bacterium *E. coli*. The minimum inhibitory concentrations (MICs) of compounds **1**–**3** were determined to be >10 mg/mL, so the new compounds had no activity against these tested pathogens.

## 3. Materials and Methods

### 3.1. General Experimental Procedures

Optical rotation was measured on a Perkin-Elmer 341 polarimeter (Perkin-Elmer, Suzhou, China). IR spectra were recorded on a Nicolet Magna FT-IR 750 spectrometer (Nicolet, Tokyo, Japan). UV spectra were recorded on a Varian CARY 300 BIO spectrophotometer (Varian, Cary, NC, USA). The HR-ESIMS spectra were taken on a Q-TOF Micro LC-MS-MS mass spectrometer (Waters Co, Milford, MA, USA). NMR spectra were measured with a Bruker DRX-400 (400 MHz for ^1^H and 100 MHz for ^13^C) spectrometer (Bruker, Rheinstetten, Germany). HPLC separation was performed on semipreparative HPLC (Agilent 1100, Zorbax SB-C18, 5 μm, 250 × 9.4 mm inner diameter; 1.5 mL/min; 220 nm; Agilent, Palo Alto, CA, USA). Column chromatography was performed with commercial silica gel (Qing Dao Hai Yang Chemical Group Co., 100–200 and 200–300 mesh). Silica gel plates HSGF254 (Yantai Chemical Industry Research Institute, Yantai, China) were used for thin-layer chromatography (TLC).

### 3.2. Isolation and Identification of Strain

Mangrove soils were collected from Zhangzhou (24°20′, 117°45′), Fujian province, China. The samples were placed into clean plastic bags and stored at 4 °C prior to isolation. Samples were dried and mixed with sterile deionized water, and actinobacteria were isolated by plating the resulting suspensions on four different selective isolation media: M6 (raffinose–histidine medium), M8 (glycerol asparagine agar medium), HV (humic acid–vitamin agar), and GP (glucose–tryptone agar medium). All of the media were supplemented with 25 mg/L of nalidixic acid and 50 mg/L of nystatin. Also, 10% supernatant of the strain *Micrococcus luteus* JCM 21373^T^, which contained resuscitation-promoting factor (rpf), was added to each medium. The strain *M. luteus* JCM 21373^T^ was inoculated in LMM (lactate minimal medium) at a rate of 5% (*v*/*v*) and incubated at 30 °C for 48 h until the end of the logarithmic growth phase [29]. Then, the culture supernatant was collected following centrifugation at 10,000 rpm and sterilized by filtration through a 0.22-μm filter. After incubation, isolates with *Micromonospora*-like morphology were transferred to ISP medium 2 (yeast extract-malt extract agar) and incubated for 7–10 days at 28 °C.

All isolates were grown in marine broth 2216 (MB, difco) medium for 5 days at 30 °C. Genomic DNA was extracted using the Bacteria Genomic DNA Extraction Kit (DongSheng Biotech) according to the manufacturer’s instructions. For phylogenetic studies, the 16S rRNA gene was amplified using two universal primers: 27F (5’-GAGTTTGATCCTGGCTCAG-3’) and 1492R (5’-AGAAAGGAGGTGATCCAGCC-3’) [30]. Amplification reactions were prepared in a 25-μL final reaction volume containing 11 μL of distilled water, 12.5 μL of PCR SuperMix, 1 μL of each primer (2.5 μM), and 0.5 μL of extracted DNA (template). PCR was performed under the following conditions: 30 cycles of 94 °C/5 min, 4 °C/30 s, 55 °C/30 s, 72 °C/75 s, and a final extension of 72 °C/10 min. PCR products were detected by agarose gel electrophoresis. The obtained sequence of each strain was assembled with DNASTAR SeqMan (LaserGene, Madison, WI). The 16S rRNA gene sequence was analyzed using the ExTaxon-e service [31] and the BLASTN program (https://blast.ncbi.nlm.nih.gov/Blast.cgi). For phylogenetic analysis, multiple sequence alignment was accomplished via the CLUSTAL W program of the MEGA 5 package [32]. The strain 110B was identified as *M. aurantiaca* because its 16S rRNA sequence (accession no: MH333275 in the GenBank) exhibited a high sequence similarity of 100% with that of *M. aurantiaca* ATCC 27029^T^ (accession no: CP002162).

### 3.3. Preparation of Crude Extracts

All strains were inoculated into 250-mL Erlenmeyer flasks containing 30 mL of MB medium and incubated at 28 °C for 7 days while shaking at 200 rpm. After cultivation, 60 mL of methanol was added to each of the cultures. After 24 h, the mixture was filtered and concentrated under vacuum. The extracts were dissolved in dimethyl sulfoxide for cytotoxic evaluation.

### 3.4. Fermentation and Extraction of Strain 110B

Strain 110B was inoculated into a 1000-mL flask containing 250 mL of seed culture medium consisting of (in %) malt extract (1.0), glucose (0.4), yeast extract (0.4), and CaCO_3_ (0.2), with a pH of 7.2–7.4. Incubation was carried out at 28 °C for 3 days on a rotary shaker operating at 250 rpm. Then, a 5.0% seed culture broth was cultured in a 50-L fermenter (containing 30 L of fermentation medium) at 28 °C for 7 days. The fermentation medium was a modified 2216 medium which contained (per liter distilled water): soybean cake (20 g), maltodextrin (10 g), peptone (5.0 g), yeast extract (10 g), glucose (10 g), NaCl (19.45 g), MgCl_2_·6H_2_O (12.6 g), MgSO_4_·7H_2_O (6.64 g), CaCl_2_ (1.8 g), KCl (0.55 g), NaHCO_3_ (0.16 g), H_3_BO_3_ (22.0 mg), KBr (0.08 g), Na_2_SiO_3_ (4.0 mg), NaF (2.4 mg), NH_4_NO_3_ (1.6 mg), Na_2_PO_4_ (8.0 mg), SrCl_2_·6H_2_O (57 mg), and ferric citrate (0.1 g).

The final 30 L of fermentation broth was centrifuged to separate the mycelial cake and supernatant. The mycelial cake was washed with water (3 L) and subsequently extracted with MeOH (3 L). The supernatant and the wash water were passed through a Diaion HP-20 resin column (Mitsubushi Chemical Co, Ltd., Tokyo, Japan) and eluted with 95% EtOH. The MeOH extract and the EtOH eluents were evaporated under reduced pressure to yield a mixture (25 g) at 50 °C. The mixture was chromatographed on a silica gel column (Qingdao Haiyang Chemical Group, Qingdao, China; 100–200 mesh) and successively eluted with CHCl_3_/MeOH (100:0, 98:2, 95:5, 90:10, 85:15, 80:20, 70:30, 60:40, and 50:50 *v*/*v*) to give six fractions (Fr.1–6) based on the TLC profiles. After the Fr.3 was concentrated in vacuo, the material was subjected to a Sephadex LH-20 gel column (GE Healthcare, Glies, UK), eluted with CHCl_3_/MeOH (1:1, *v*/*v*), and detected using TLC to give three fractions (Fr.3-1–3-3). Fr.3-2 was further analyzed and purified by semipreparative reversed-phase HPLC eluting with CH_3_CN/H_2_O (30:70, *v*/*v*) to give compounds **1** (*t*_R_ 15.5 min, 12.2 mg) and **2** (*t*_R_ 13.5 min, 10.1 mg). Fr.4 was subjected to a Sephadex LH-20 column eluted with CHCl_3_/MeOH (1:1, *v*/*v*) and detected by TLC to give three subfractions (Fr.4-1–4-3). Fr.4-2 was further separated by semipreparative reversed-phase HPLC eluting with CH_3_CN/H_2_O (30:70, *v*/*v*) to give compound **3** (*t*_R_ 10.6 min, 8.9 mg).

Compound **1**: white powder; [*α*${]}_{D}^{25}$−107 (*c* 0.04, EtOH); IR (KBr) *ν*_max_ 3663, 3553, 2984, 2905, 1628, 1407, 1049 cm^−1^; UV (EtOH) *λ*_max_ (log *ε*) 249 (4.49), 301 (4.10) nm; ^1^H (400 MHz) and ^13^C NMR (100 MHz) data, see Table 2; positive HR-ESIMS *m/z* 385.1286 [M + H]^+^ (calcd. for C_21_H_21_O_7_, 385.1282).

Compound **2**: white powder; [*α*${]}_{D}^{25}$−112 (*c* 0.04, EtOH); IR (KBr) *ν*_max_ 3357, 2984, 2838, 1634, 1408, 1023 cm^−1^; UV (MeOH) *λ*_max_ (log *ε*) 249 (4.18), 262 (4.23), 305 (3.76) nm; ^1^H (400 MHz) and ^13^C NMR (100 MHz) data, see Table 2; positive HR-ESIMS *m/z* 385.1287 [M + H]^+^ (calcd. for C_21_H_21_O_7_, 385.1282).

Compound **3**: white powder; [*α*${]}_{D}^{25}$−27 (*c* 0.04, EtOH); IR (KBr) *ν*_max_ 3381, 2987, 1650, 1450, 1407, 1022 cm^−1^; UV (MeOH) *λ*_max_ (log *ε*) 249 (4.16), 259 (4.23), 305(3.81) nm; ^1^H (400 MHz) and ^13^C NMR (100 MHz) data, see Table 2; positive HR-ESIMS *m/z* 515.1912 [M + H]^+^ (calcd. for C_27_H_31_O_10_, 515.1912).

### 3.5. Determination of Aglycone Moieties and Sugars Configuration

Compounds **1** (2.0 mg), **2** (2.0 mg), and **3** (1.5 mg) were refluxed with 2 mL of 2 N HCl and heated for 2 h at 80 °C. After being neutralized with NaHCO_3,_ the hydrolysate was extracted with EtOAc to separate the organic and aqueous layers. The organic layer was dried in vacuo and subjected to column chromatography using a Zorbax B-C18 column, mobile phase of CH_3_CN/H_2_O (30:70, *v*/*v*), flow rate of 1.5 mL/min^−1^, and detection wavelength at 254 nm. HPLC analysis of the aglycone-containing fractions of **1**–**3** gave peaks at 22.34, 22.38, and 22.46 min, respectively, while the *t_R_* values for standard daidzein were observed at 22.47 min, suggesting that the aglycone moieties of **1**–**3** were daidzein. The aqueous fraction was evaporated in vacuo and the sugar residue was dissolved in pyridine (2 mL) containing l-cysteine methyl ester hydrochloride (1.0 mg), followed by heating at 60 °C for 1 h. A 25-μL solution of *O*-tolylisothiocyanate was added to the mixture, which was heated at 60 °C for a further 1 h [33]. Likewise, the *O*-tolylthiocarbamate derivative of 2-deoxy-l-fucose was prepared according to the method described above. Then, the *O*-tolylthiocarbamate derivatives were analyzed by reversed-phase HPLC (Amethyst C18-H, 5 μm, 250 × 4.6 mm inner diameter; 0.8 mL/min^−1^; 250 nm) eluting with CH_3_CN/H_2_O (30:70, *v*/*v*). Under these conditions, the derivative of standard sugar gave a peak at *t*_R_ (min) = 25.06, while *O*-tolylthiocarbamate derivatives of the liberated sugars of **1**–**3** showed peaks at 25.40, 25.02, and 25.02 min, respectively. The sugar moieties of the three new compounds (**1**–**3**) were identified as 2-deoxy-l-fucose.

### 3.6. Biological Assays

The antimicrobial activity of the three compounds against pathogenic fungi *C. albicans*, pathogenic bacteria methicillin-resistant *S. aureus*, and *E. coli* was investigated with the minimum inhibitory concentration (MIC) method recommended by the Clinical and Laboratory Standards Institute [34]. Gentamicin (an antibacterial antibiotic) and amphotericin B (an antifungal antibiotic) were used as a positive control.

The cytotoxicity of the three compounds was assayed in vitro against the human lung carcinoma cell line A549, hepatocellular liver carcinoma cell line HepG2, and the human colon tumor cell line HCT116 by the cell counting kit-8 (CCK8) colorimetric method. The cell lines were cultured in Dulbecco’s Modified Eagle’s Medium (DMEM) containing 10% calf serum at 37 °C for 4 h in a 5% CO_2_ incubator. The adherent cells of the logarithmic growth stage were digested and seeded in a 96-well culture plate at a density of 1 × 10^4^ cells per/well. Test samples and control were added to the medium and incubated for 48 h. Then, the cell counting kit-8 (CCK-8, Dojindo, Kumamoto, Japan) reagent was added to the medium and incubated for 3 h. Cell viability was measured by absorbance at 450 nm using a SpectraMax M5 microplate reader (Molecular Devices Inc., Sunnyvale, CA, USA) [35]. The inhibitory rate of cell proliferation was expressed as IC_50_ values. Doxorubicin was used as a positive control, and cell solutions containing 0.5% DMSO were tested as a negative control.

## 4. Conclusions

The study was designed to isolate mangrove-derived *Micromonospora* strains and explore their bioactive metabolites. Fifty-one strains belonging to the genus *Micromonospora* were isolated, and the crude extracts of 12 isolates showed cytotoxic activity against the human lung carcinoma cell line A549. Furthermore, a chemical investigation was carried out on the strain *M. aurantiaca* 110B. This investigation led to the isolation of three new isoflavonoid glycosides (compounds **1**–**3**). The structures of the new compounds were determined by NMR, HR-ESIMS, acid hydrolysis, and HPLC analysis on the *O*-tolylthiocarbamate derivatives of sugar moieties. Moreover, the three new compounds were the result of biotransformation by the strain *M. aurantiaca* 110B. The three compounds showed moderate activity against the human lung carcinoma cell line A549, hepatocellular liver carcinoma cell line HepG2, and the human colon tumor cell line HCT116.

## Figures and Tables

**Table 1:** ^1^H and ^13^C NMR (100 MHz) data of compounds **1**−**3** in CD_3_OD.

Position	1^a^		2^b^		3^b^	
	*δ*c	*δ*_H_ (*J* in Hz)	*δ*c	*δ*_H_ (*J* in Hz)	*δ*c	*δ*_H_ (*J* in Hz)
2	154.9	8.08 s	154.9	8.12 s	155.2	8.21 s
3	125.6		126.2		125.9	
4	178		178.1		177.9	
5	128.5	7.97 d (9.0)	128.2	8.06 d (9.0)	128.2	8.12 d (8.9)
6	116.5	6.84 dd (9.0, 2.4)	117.2	7.09 dd (9.0, 2.3)	117.2	7.15 dd (8.9, 2.2)
7	164.7		163.1		163.1	
8	103.3	6.76 d (2.4)	104.4	7.18 d (2.3)	104.4	7.25 d (2.2)
9	159.8		159.4		159.3	
10	118.2		119.8		119.7	
1′	126.7		124.1		125.9	
2′, 6′	131.3	7.39 d (8.7)	131.4	7.31 d (9.0)	131.3	7.47 d (8.7)
3′, 5′	117.5	7.05 d (8.7)	116.3	6.78 d (9.0)	117.4	7.13 d (8.7)
4′	158.3		158.8		158.3	
1″	97.6	5.62 br d (3.5)	98.2	5.78 br d (3.6)	97.6	5.69 br d (3.5)
2″	32.1	2.03 ddd (12.8, 11.9, 3.6)	32.9	2.07 ddd (13.2,12.0, 3.6)	33.2	2.08 ddd (12.8, 12.0, 3.5)
		1.90 dd (12.8, 5.0)		1.96 dd (13.2, 5.0)		1.91 dd (12.8, 5.1)
3″	66.7	4.09 ddd (11.9, 5.0, 3.0)	66.6	4.09 ddd (12.0, 5.0, 3.0)	66.7	4.16 ddd (12.0, 5.1, 3.1)
4″	72.1	3.55 br d (3.0)	71.9	3.57 br d (3.0)	71.9	3.62 m
5″	68.6	3.89 q (6.6)	69.3	3.86 q (6.6)	69.3	3.91 m
6″	18.4	1.10 d (6.6)	17.2	1.12 d (6.6)	17.2	1.18 d (6.2)
1‴					98.2	5.84 br d (3.5)
2‴					32.9	2.12 ddd (13.1, 12.0, 3.5)
						1.98 dd (13.1, 5.1)
3‴					66.5	4.13 ddd (12.0, 5.1, 3.1)
4‴					72.1	3.62 m
5‴					68.6	3.95 m
6‴					17.2	1.16 d (6.2)

^a 1^H (600 MHz); ^b 1^H (400 MHz).

**Table 2:** Cytotoxic activity of compounds **1**–**3** against selected human tumor cell lines.

Compounds	IC_50_ (μg/mL)		
	A549	HepG2	HCT116
**1**	17.55	52.71	16.00
**2**	16.95	50.90	15.40
**3**	14.79	44.42	13.48
**Doxorubicin**	1.02	0.86	0.91

## Supplementary Materials

The following are available online https://www.mdpi.com/1660-3397/17/5/294/s1: HR-ESIMS and NMR spectra (Figures S1–S29) of compounds **1**–**3**, as well as other supporting data.
