# Supplementary material for: Three New Isoflavonoid Glycosides from the Mangrove-Derived Actinomycete Micromonospora aurantiaca 110B
Source: Mar Drugs. 2019 May 17;17(5):294. doi: 10.3390/md17050294 (PMC6562861; doi:10.3390/md17050294)
Supplement: Supplementary file 1 [file marinedrugs-17-00294-s001.pdf]

marine drugs

Supplementary Materials

Three New Isoflavonoid Glycosides from Mangrove-Derived Actinomycete *Micromonospora aurantiaca* 110B

Rui-Jun Wang [1], Shao-Yong Zhang [3], Yang-Hui Ye [1], Zhen Yu [3], Huan Qi [3], Hui Zhang [2,3], Zheng-Lian Xue [2], Ji-Dong Wang [2,3,] * and Min Wu [1,] *

1 Ocean College, Zhejiang University, Zhoushan 316021, China; 15034663445@163.com (R.-J.W.); 1593591913@qq.com (Y.-H.Y.)
2 College of Biochemical Engineering, Anhui Polytechnic University, Wuhu 241000, China; xuezhen0851@sina.com (Z.-L.X.)
3 Zhejiang Key Laboratory of Antifungal Drugs, Zhejiang Hisun Pharmaceutical Co., Ltd., Taizhou 318000, China; 1zhangshaoyong@163.com (S.-Y.Z.); yuzhen@hisunpharm.com (Z.Y.); qihuan@hisunpharm.com (H.Q.); huizhang@hisunpharm.com (H.Z.)
* Correspondence: jdwang@hisunpharm.com (J.-D.W.); wumin@zju.edu.cn (M. W); Tel.: +86-136-3400-5685 (J.-D.W.); +86-136-1651-0066 (M.W.)

Contents

Figure S1. HR-ESIMS data of compound **1** 3

Figure S2. The IR spectrum of compound **1** 4

Figure S3. ^{1}H NMR spectrum of compound **1** in CD_3OD (600 MHz) 4

Figure S4. ^{13}C NMR spectrum of compound **1** in CD_3OD (400 MHz) 5

Figure S5. The DEPT spectrum of compound **1** in CD_3OD (400 MHz) 5

Figure S6. The HSQC spectrum of compound **1** in CD_3OD (400 MHz) 6

Figure S7. The ^{1}H-^{1}H COSY spectrum of compound **1** in CD_3OD (400 MHz) 6

Figure S8. The HMBC spectrum of compound **1** in CD_3OD (400 MHz) 7

Figure S9. The NOESY spectrum of compound **1** in CD_3OD (600 MHz) 7

Figure S10. HR-ESIMS data of compound **2** 8

Figure S11. The IR spectrum of compound **2** 9

Figure S12. The ^{1}H NMR spectrum of compound **2** in CD_3OD (400 MHz) 9

Figure S13. The ^{13}C NMR spectrum of compound **2** in CD_3OD (400 MHz) 10

Figure S14. The DEPT spectrum of compound **2** in CD_3OD (400 MHz) 10

Figure S15. The HSQC spectrum of compound **2** in CD_3OD (400 MHz) 11

Figure S16. The ^{1}H-^{1}H COSY spectrum of compound **2** in CD_3OD (400 MHz) 11

Figure S17. The HMBC spectrum of compound **2** in CD_3OD (400 MHz) 12

Figure S18. The NOESY spectrum of compound **2** in CD_3OD (600 MHz) 12

Figure S19. HR-ESIMS data of compound **3** 13

Figure S20. The IR spectrum of compound **3** 14

Figure S21. ^{1}H NMR spectrum of compound **3** in CD_3OD (400 MHz) 14

Figure S22. ^{13}C NMR spectrum of compound **3** in CD_3OD (400 MHz) 15

Figure S23. The DEPT spectrum of compound **3** in CD_3OD (400 MHz) 15

Figure S24. The HSQC spectrum of compound **3** in CD_3OD (400 MHz) 16

Figure S25. The ^{1}H-^{1}H COSY spectrum of compound **3** in CD_3OD (400 MHz) 16

Figure S26. The HMBC spectrum of compound **3** in CD_3OD (400 MHz) 17

Figure S27. The NOESY spectrum of compound **3** in CD_3OD (600 MHz) 17

Figure S28. The retention time of the aglycone moieties of daidzein and compounds **1-3** 18

Figure S29. The retention time of the *O*-tolylthiocarbamate derivatives of 2-deoxy-L-fucose and the liberated sugars of compounds **1-3** 18

Best	ID Sourc	Formula	Species	m/z	Score	Diff (ppm)	Score (M	Mass (MFG)
	MFG	C21 H20 O7	(M+H)+	385.1286	98.97	-0.95	98.97	384.1209

Species	m/z	Score (iso. abund)	Score (mass)	Score (MFG,	Score (MS)	Score (MFG)	Score (iso	Height	Ion Formula
(M+H)+	385.1286	98.08	99.24		98.97	98.97	99.5	801604.1	C21 H21 O7

Height (C	Height S	Height % (Calc)	m/z (Calc)	Diff (mDa)	Height	Height %	Height Su	m/z	Diff (ppm)
787123.6	78.3	100	385.1282	-0.4	801604.1	100	79.7	385.1286	-1.08
182779.6	18.2	23.2	386.1316	-0.1	173537.8	21.6	17.3	386.1317	-0.23
31577.2	3.1	4	387.1341	-0.6	26483.1	3.3	2.6	387.1346	-1.51
4050	0.4	0.5	388.1367	-0.6	3905.4	0.5	0.4	388.1373	-1.43

Best	ID Sourc	Formula	Species	m/z	Score	Diff (ppm)	Score (M	Mass (MFG)
	MFG	C20 H14 N7 O2	(M+H)+	385.1286	97.47	-1.32	97.47	384.1209
	MFG	C22 H16 N4 O3	(M+H)+	385.1286	94.88	2.35	94.88	384.1222
	MFG	C19 H18 N3 O6	(M+H)+	385.1286	92	-4.62	92	384.1196
	MFG	C14 H20 N6 O5 S	(M+H)+	385.1286	90.2	-0.12	90.2	384.1216
	MFG	C18 H12 N10 O	(M+H)+	385.1286	89.39	-5.01	89.39	384.1196
	MFG	C15 H16 N10 O S	(M+H)+	385.1286	88.74	3.16	88.74	384.1229

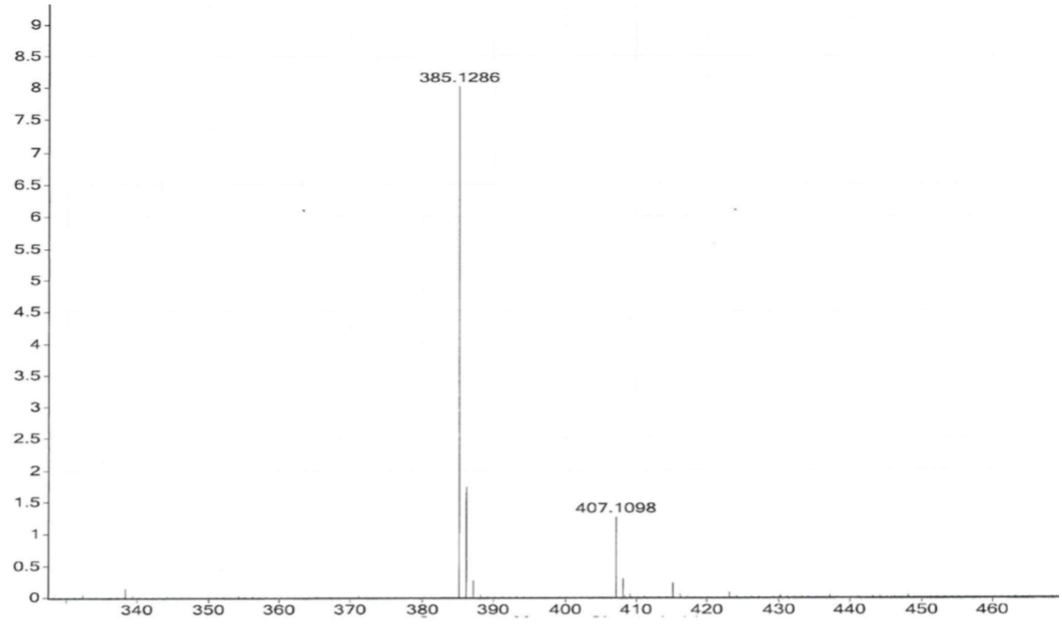

Figure S1. HR-ESIMS data of compound **1**

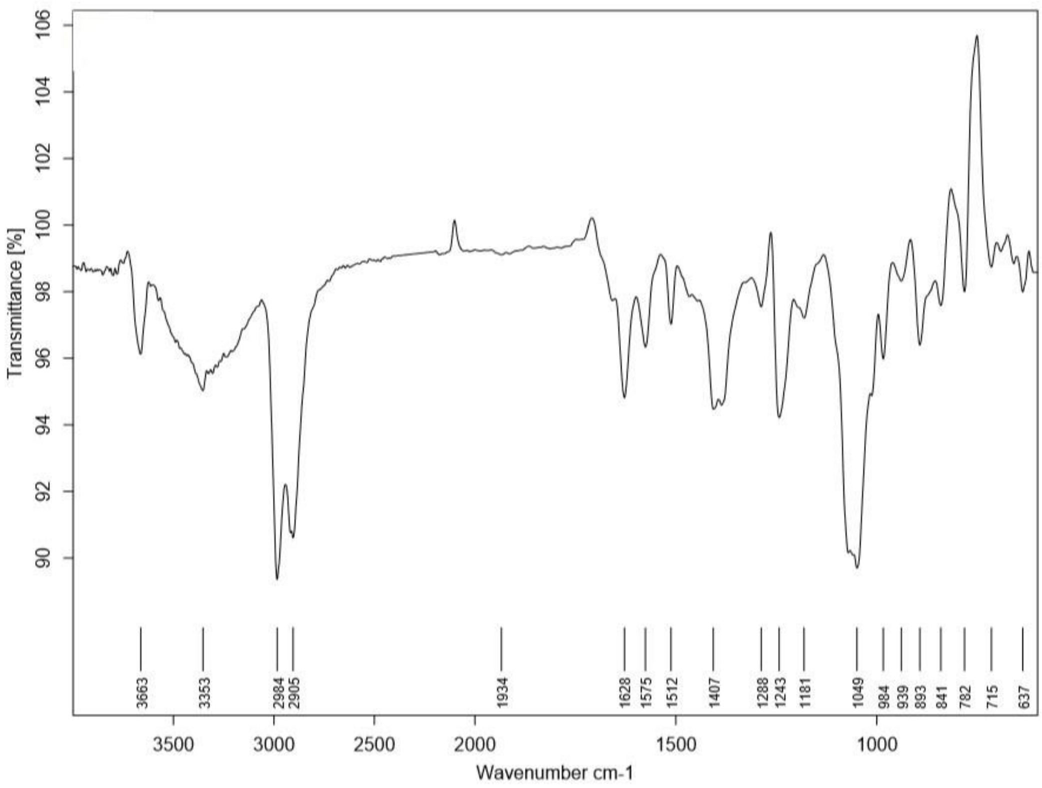

Figure S2. The IR spectrum of compound **1**

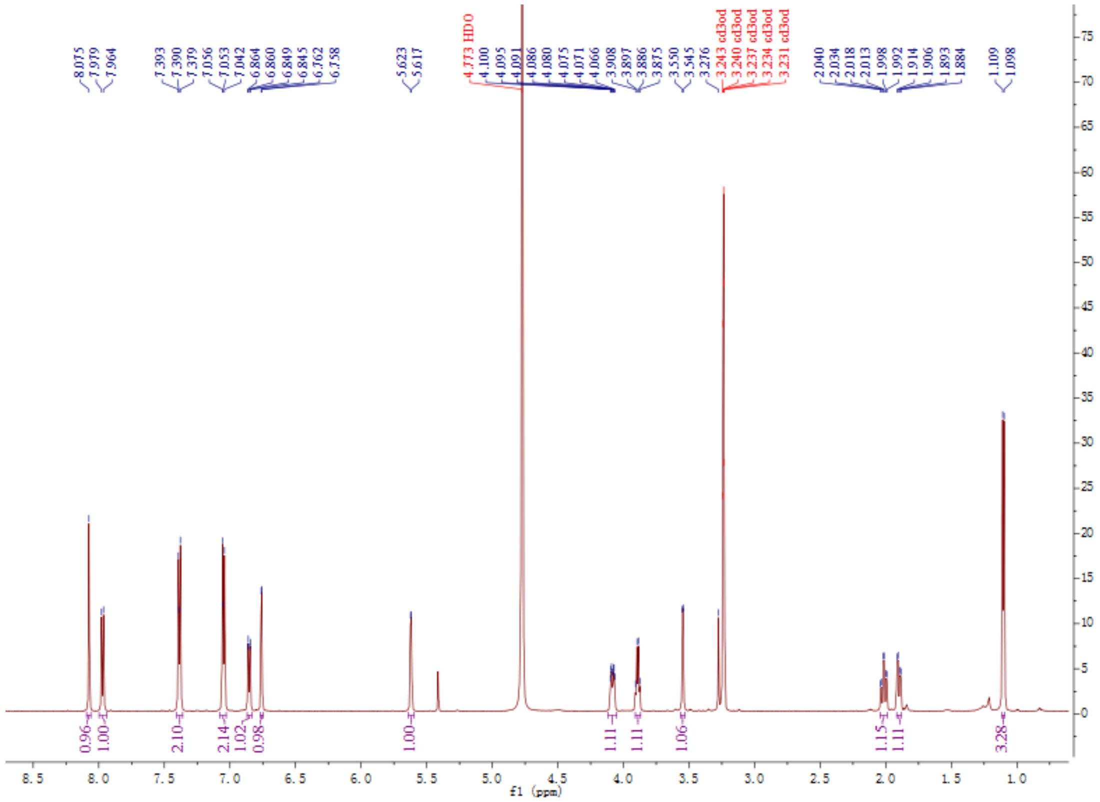

Figure S3. ^{1}H NMR spectrum of compound **1** in CD_3OD (600 MHz)

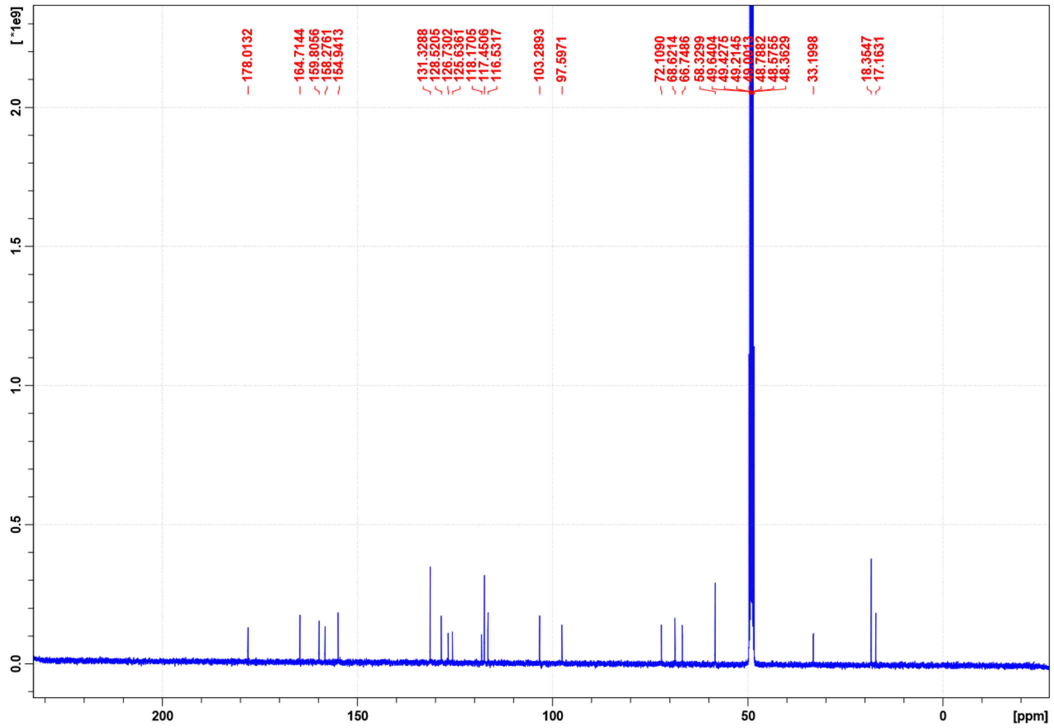

Figure S4. ^{13}C NMR spectrum of compound **1** in CD_3OD (400 MHz)

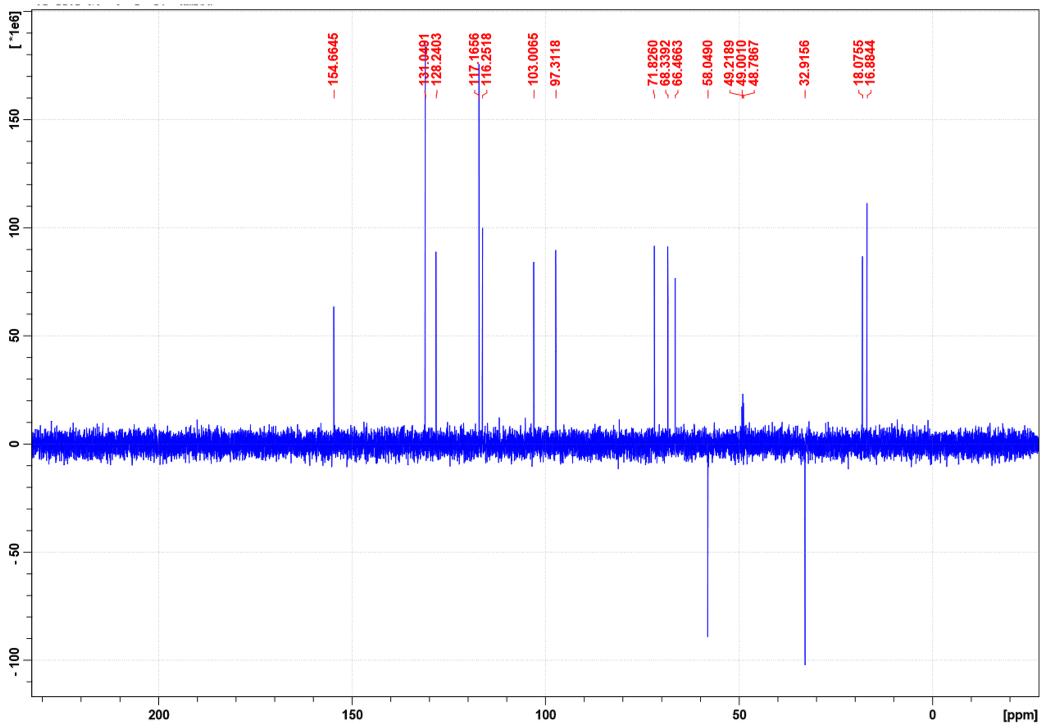

Figure S5. The DEPT spectrum of compound **1** in CD_3OD (400 MHz)

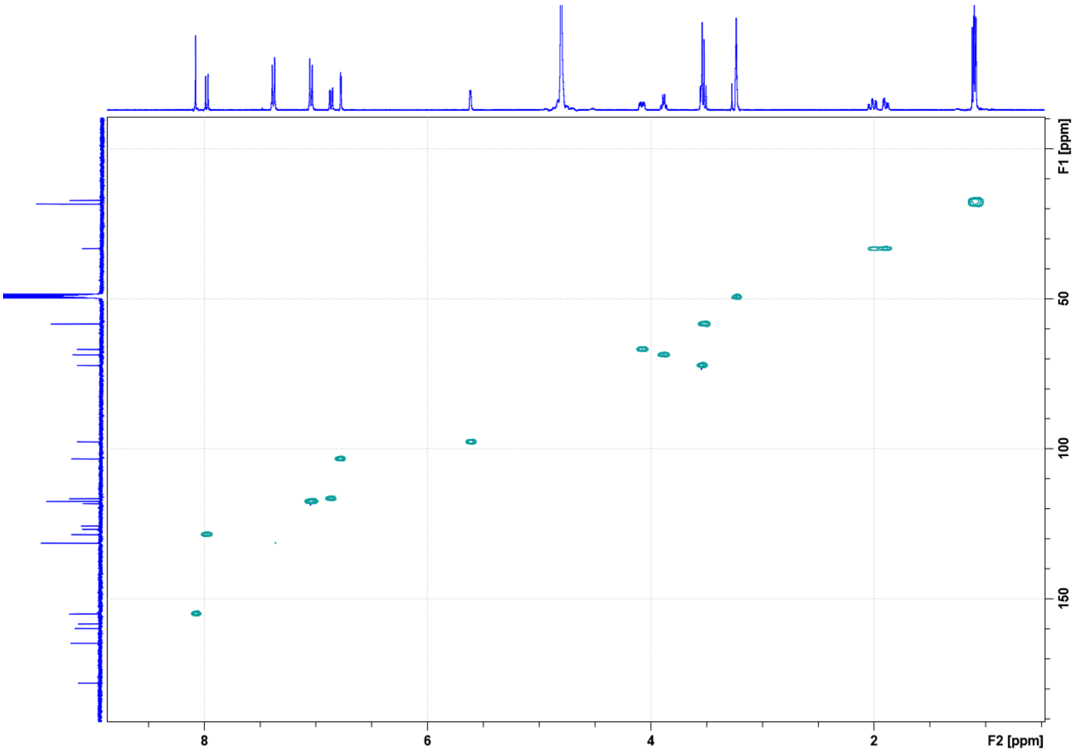

Figure S6. The HSQC spectrum of compound **1** in CD_3OD (400 MHz)

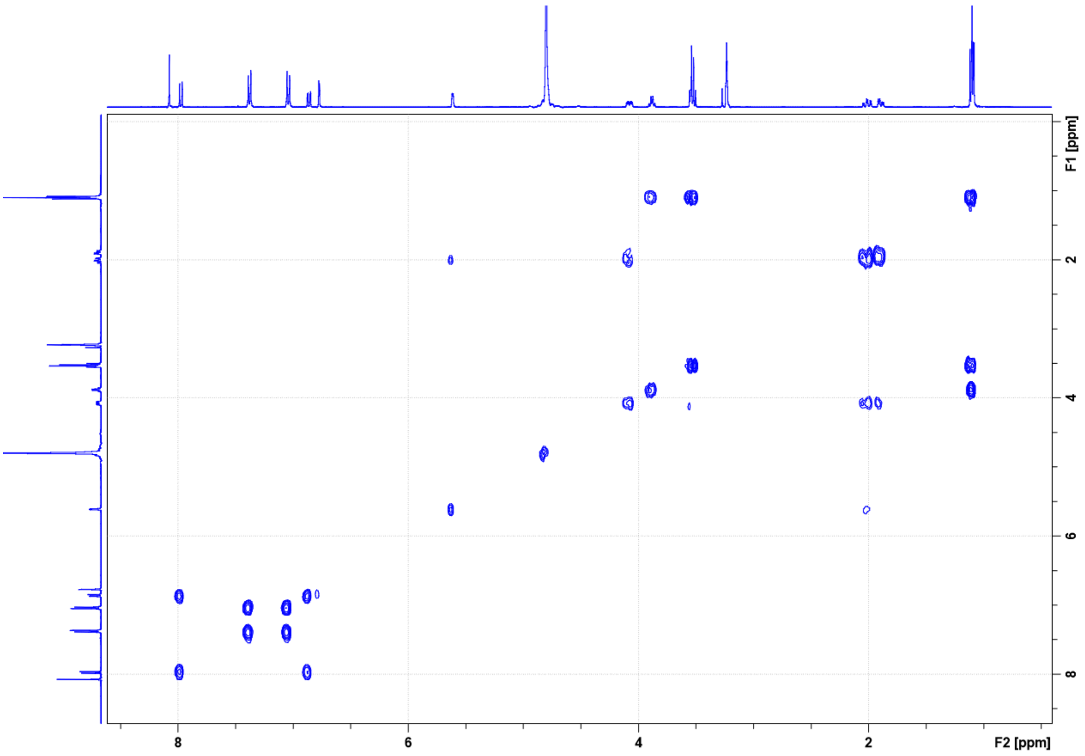

Figure S7. The ^1H-^1H COSY spectrum of compound **1** in CD_3OD (400 MHz)

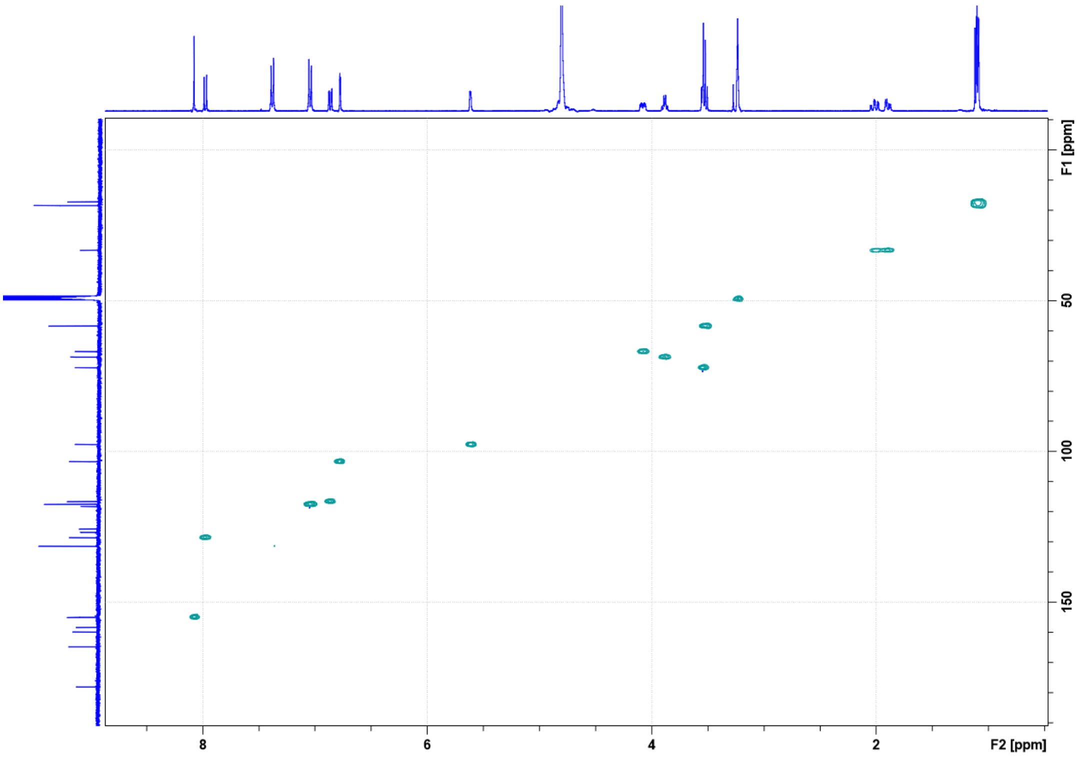

Figure S8. The HMBC spectrum of compound **1** in CD_3OD (400 MHz)

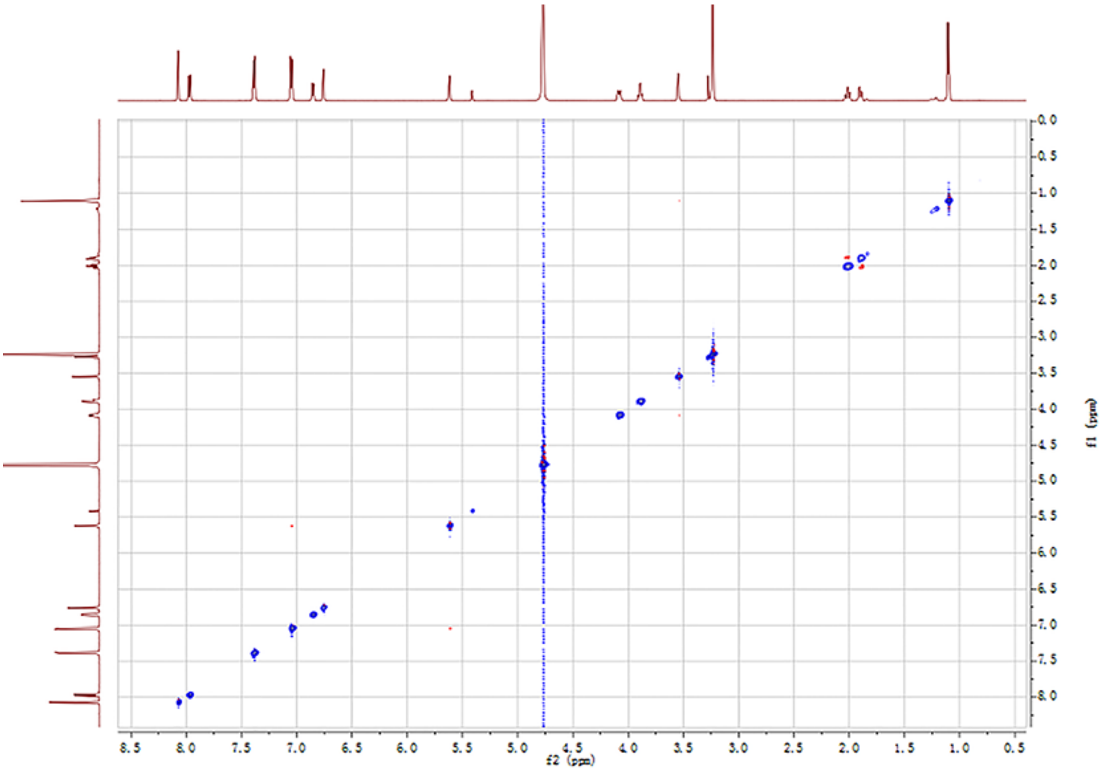

Figure S9. The NOESY spectrum of compound **1** in CD_3OD (600 MHz)

Spectrum Identification Results: + Scan (rt: 0.165 min) Sub (110B-N7.d)

Best	ID Source	Formula	Species	m/z	Score	Diff (ppm)	Score (MFG)	Mass (MFG)
	MFG	C21 H20 O7	(M+H)+	385.1287	98.45	-1.27	98.45	384.1209

Species	m/z	Score (iso. abund)	Score (mass)	Score (MFG,	Score (MS)	Score (MFG)	Score (iso. spec	Height	Ion Formula
(M+H)+	385.1287	97.71	98.64		98.45	98.45	98.98	506509.2	C21 H21 O7

Height (Calc)	Height S	Height % (Calc)	m/z (Calc)	Diff (mDa)	Height	Height %	Height Sum %	m/z	Diff (ppm)
497440.6	78.3	100	385.1282	-0.6	506509.2	100	79.7	385.1287	-1.43
115511.7	18.2	23.2	386.1316	-0.1	108520.3	21.4	17.1	386.1317	-0.21
19955.9	3.1	4	387.1341	-0.5	17854.8	3.5	2.8	387.1346	-1.34
2559.5	0.4	0.5	388.1367	-4.8	2583.3	0.5	0.4	388.1415	-12.37

Best	ID Source	Formula	Species	m/z	Score	Diff (ppm)	Score (MFG)	Mass (MFG)
	MFG	C20 H14 N7 O2	(M+H)+	385.1287	96.83	-1.64	96.83	384.1209
	MFG	C22 H16 N4 O3	(M+H)+	385.1287	95.03	2.03	95.03	384.1222
	MFG	C19 H18 N3 O6	(M+H)+	385.1287	90.91	-4.94	90.91	384.1196
	MFG	C14 H20 N6 O5 S	(M+H)+	385.1287	90.9	-0.47	90.9	384.1216
	MFG	C15 H16 N10 O S	(M+H)+	385.1287	90.18	2.81	90.18	384.1229
	MFG	C18 H12 N10 O	(M+H)+	385.1287	88.2	-5.33	88.2	384.1196

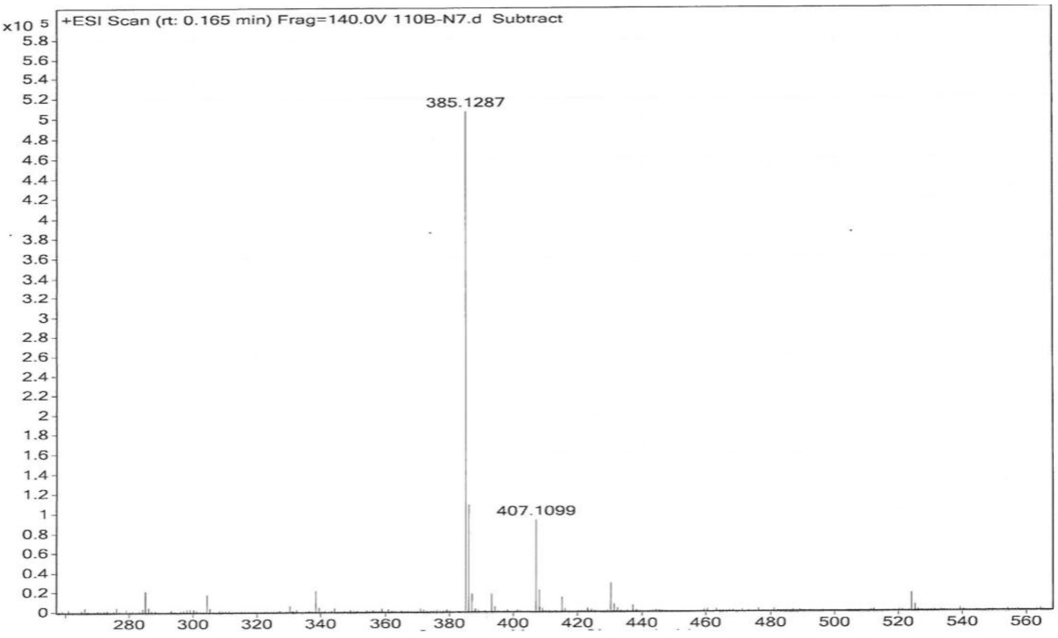

Figure S10. HR-ESIMS data of compound **2**

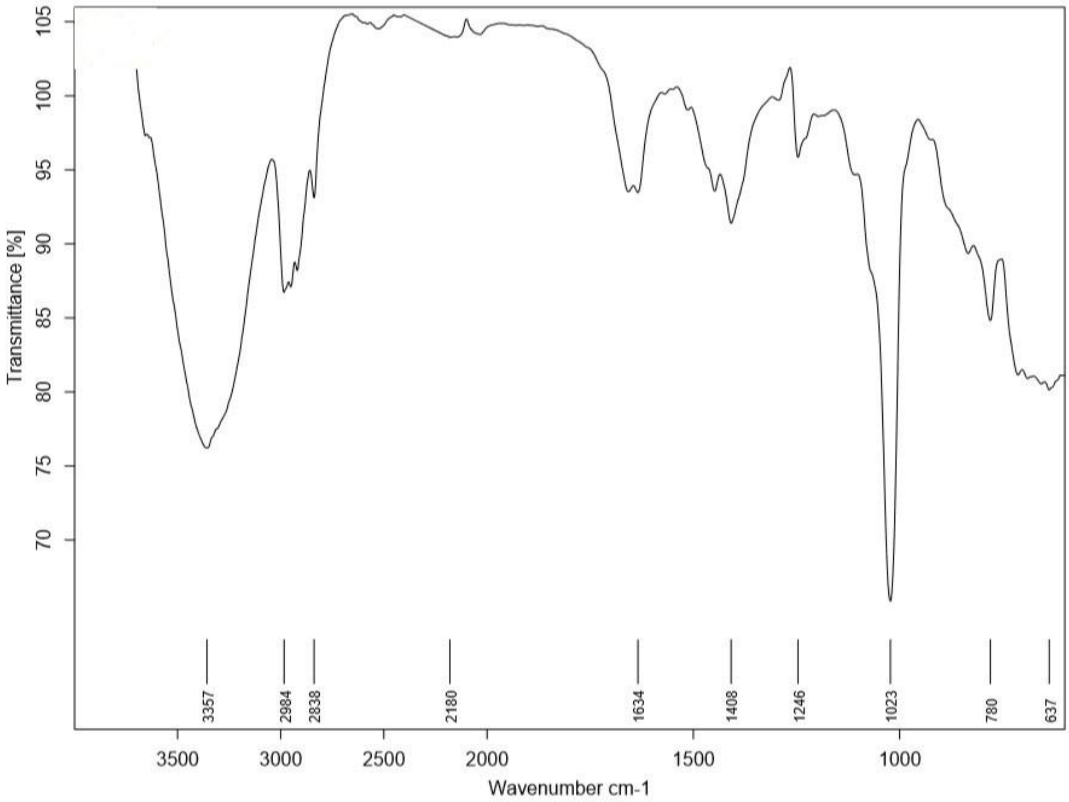

Figure S11. The IR spectrum of compound **2**

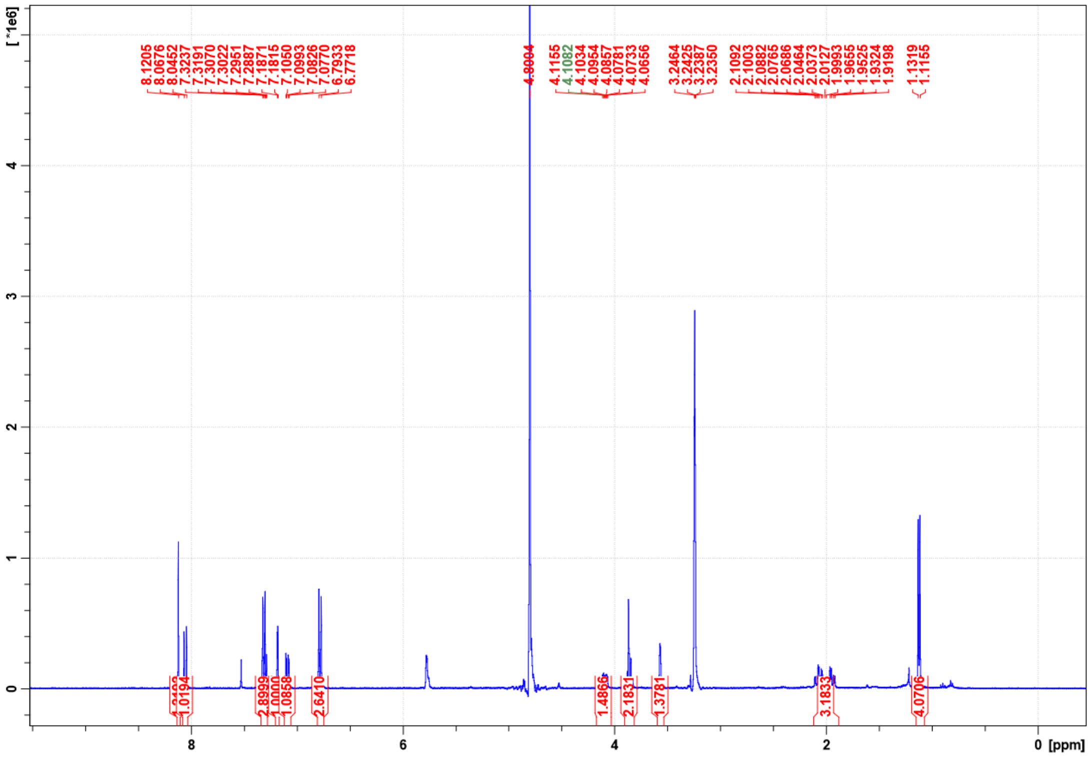

Figure S12. The ^{1}H NMR spectrum of compound **2** in CD_3OD (400 MHz)

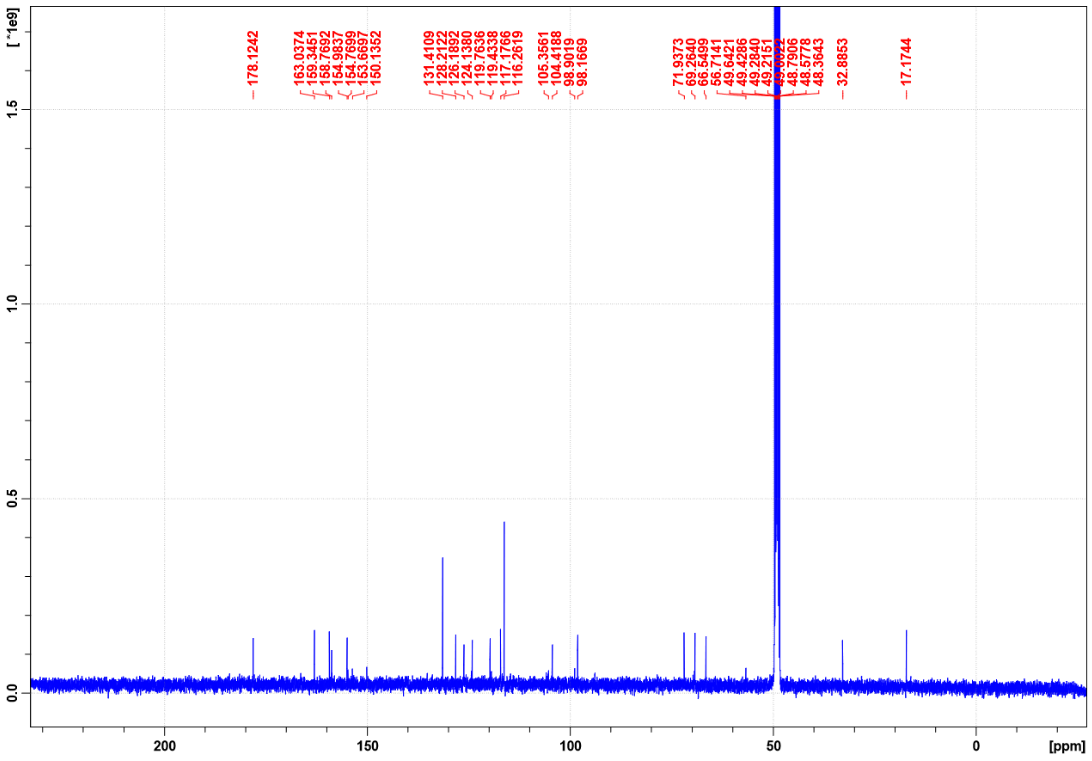

Figure S13. The ^{13}C NMR spectrum of compound **2** in CD_3OD (400 MHz)

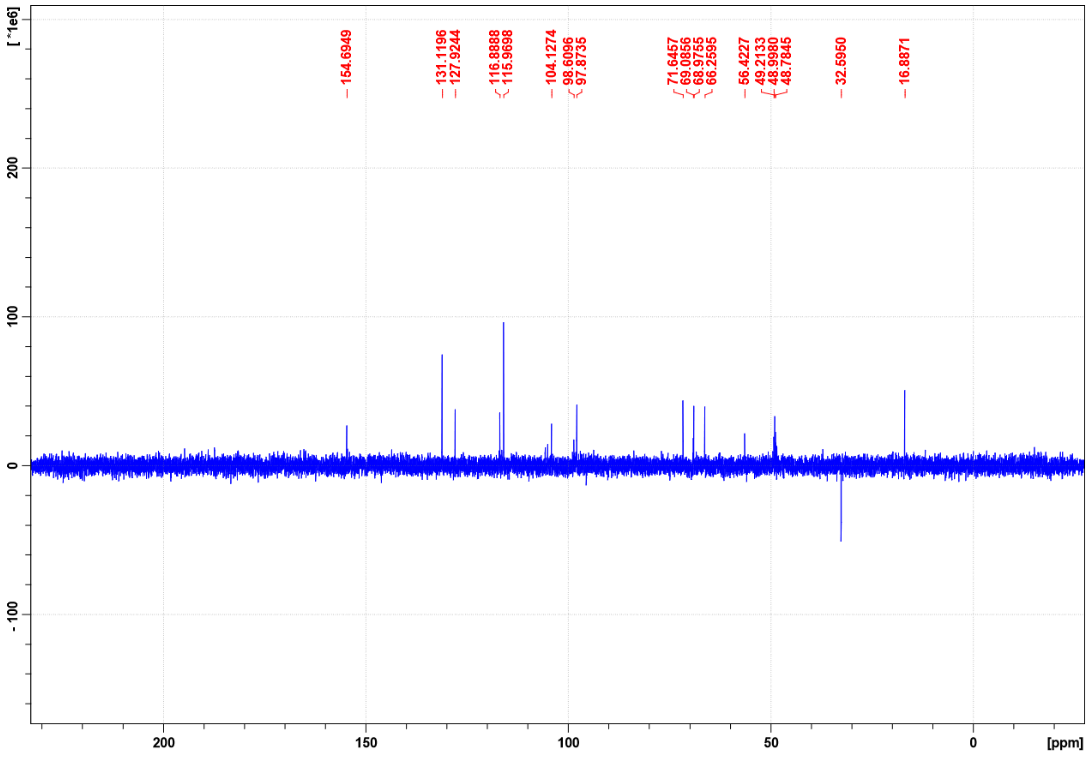

Figure S14. The DEPT spectrum of compound **2** in CD_3OD (400 MHz)

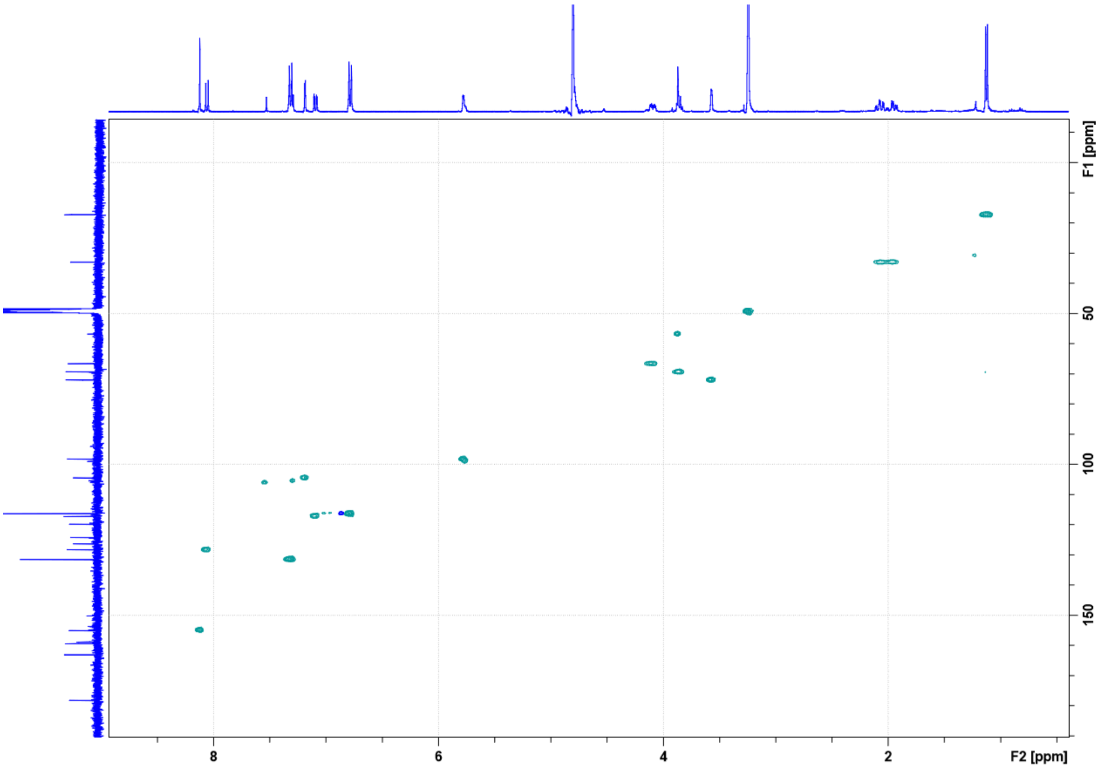

Figure S15. The HSQC spectrum of compound **2** in CD_3OD (400 MHz)

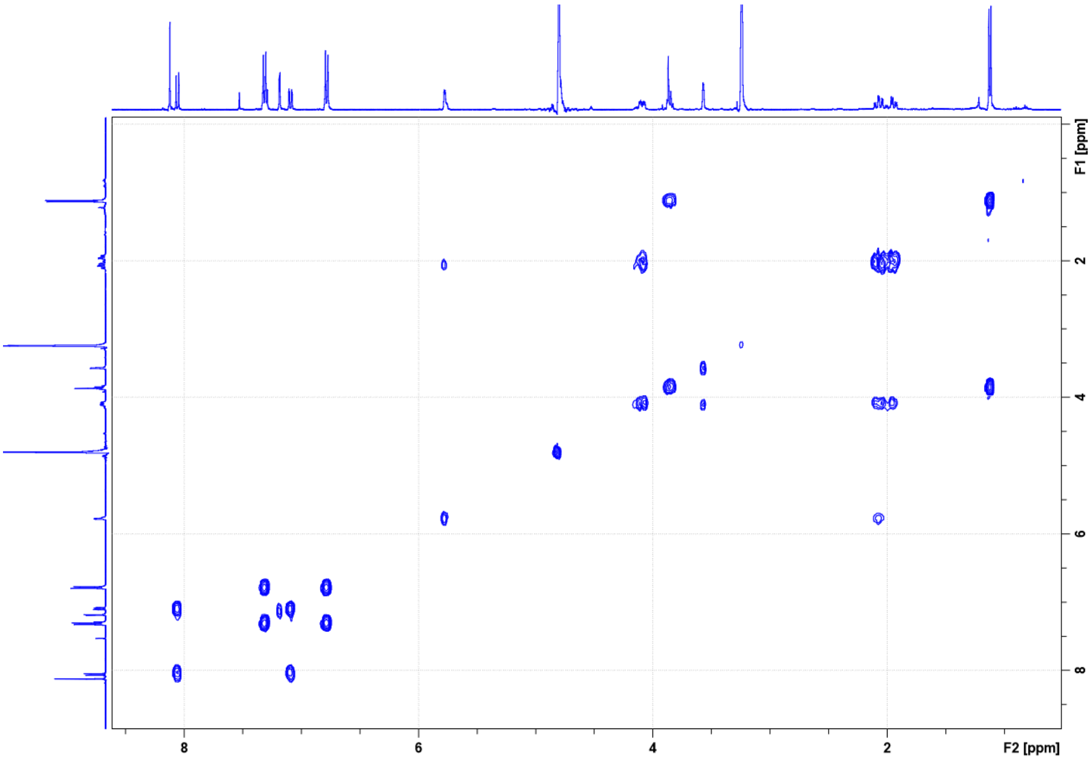

Figure S16. The ^1H-^1H COSY spectrum of compound **2** in CD_3OD (400 MHz)

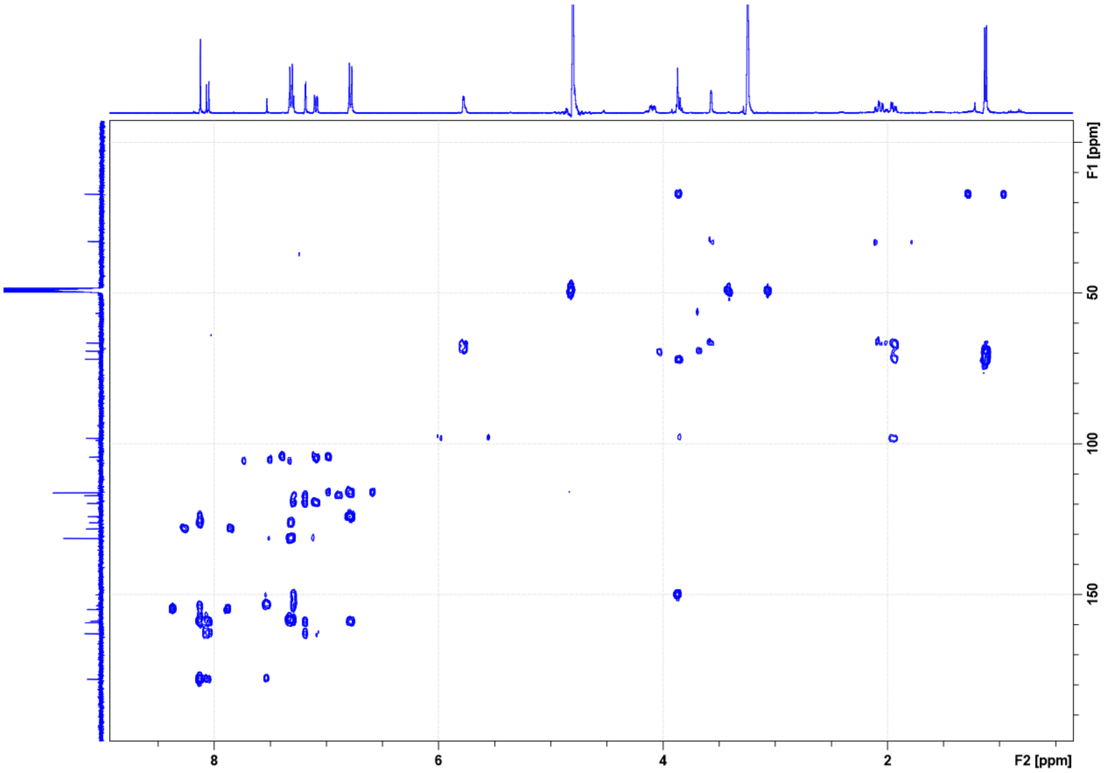

Figure S17. The HMBC spectrum of compound **2** in CD_3OD (400 MHz)

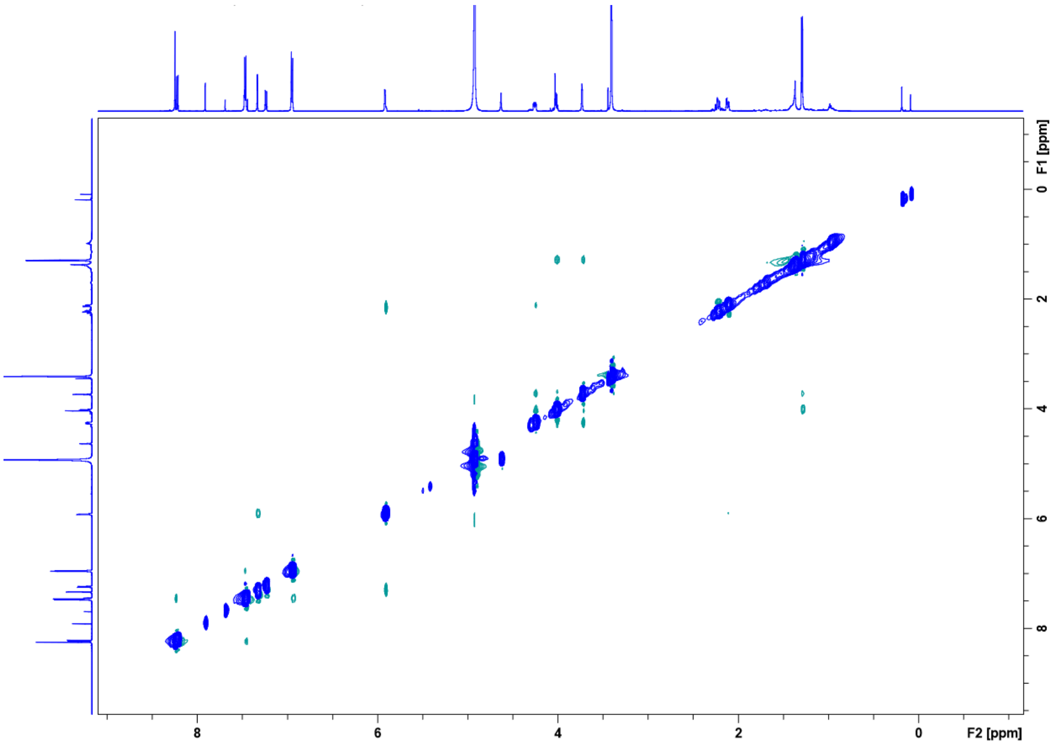

Figure S18. The NOESY spectrum of compound **2** in CD_3OD (600 MHz)

Spectrum Identification Results: + Scan (rt: 0.163-0.180 min) Sub (110B-N2.d)

Best	ID Sourc	Formula	Species	m/z	Score	Diff (ppm)	Score (M	Mass (MFG)
	MFG	C27 H30 O10	(M+H)+	515.1912	99.66	0.14	99.66	514.1839

Species	m/z	Score (iso. abund)	Score (mass)	Score (MFG,	Score (MS)	Score (MFG)	Score (iso	Height	Ion Formula
(M+H)+	515.1912	99.68	99.98		99.66	99.66	98.98	441638.1	C27 H31 O10

Height (C	Height S	Height % (Calc)	m/z (Calc)	Diff (mDa)	Height	Height %	Height Su	m/z	Diff (ppm)
437973.4	72.7	100	515.1912	0	441638.1	100	73.4	515.1912	-0.09
131129	21.8	29.9	516.1946	0.4	129263.9	29.3	21.5	516.1941	0.85
27938.2	4.6	6.4	517.1972	0.6	26185	5.9	4.3	517.1965	1.17
4448.1	0.7	1	518.1998	-2.1	4485.5	1	0.7	518.202	-4.1
589	0.1	0.1	519.2024	3.6	505.2	0.1	0.1	519.1988	6.92

Best	ID Sourc	Formula	Species	m/z	Score	Diff (ppm)	Score (M	Mass (MFG)
	MFG	C26 H24 N7 O5	(M+H)+	515.1912	99.29	-0.15	99.29	514.1839
	MFG	C25 H18 N14	(M+H)+	515.1912	97.4	-0.44	97.4	514.1839
	MFG	C25 H28 N3 O9	(M+H)+	515.1912	96.46	-2.6	96.46	514.1826
	MFG	C24 H22 N10 O4	(M+H)+	515.1912	95.29	-2.9	95.29	514.1825
	MFG	C28 H26 N4 O6	(M+H)+	515.1912	95.12	2.6	95.12	514.1852
	MFG	C27 H20 N11 O	(M+H)+	515.1912	94.11	2.32	94.11	514.1852
	MFG	C19 H24 N13 O3 S	(M+H)+	515.1912	92.47	0.28	92.47	514.1846
	MFG	C20 H30 N6 O8 S	(M+H)+	515.1912	90.66	0.63	90.66	514.1846
	MFG	C21 H26 N10 O4 S	(M+H)+	515.1912	88.92	3.07	88.92	514.1859
	MFG	C34 H28 N O2 S	(M+H)+	515.1912	88.51	0.03	88.51	514.1841

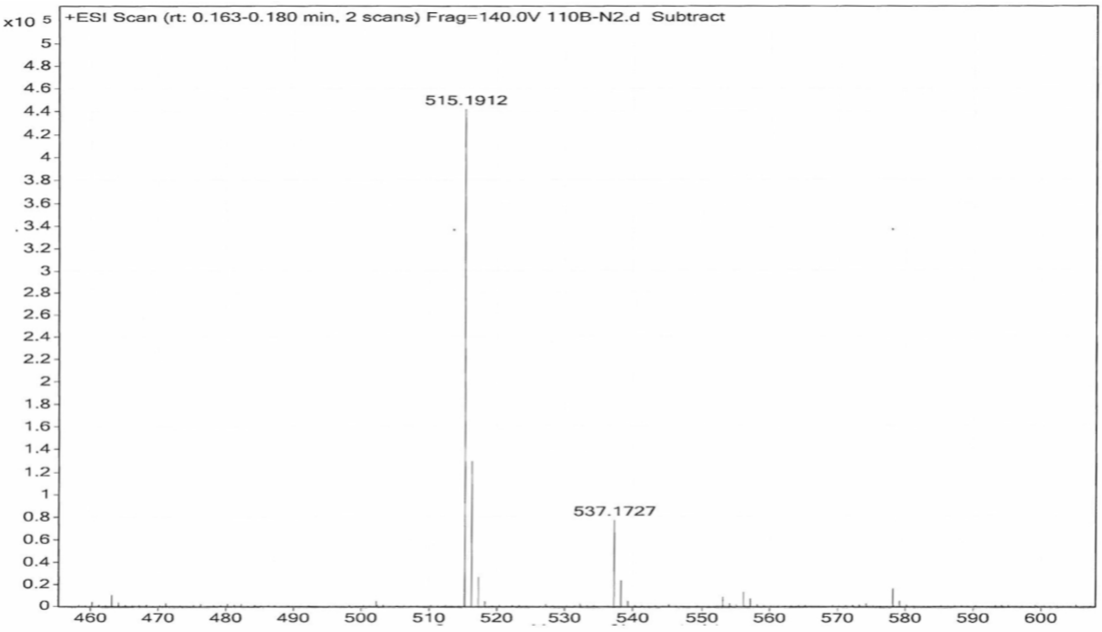

Figure S19. HR-ESIMS data of compound **3**

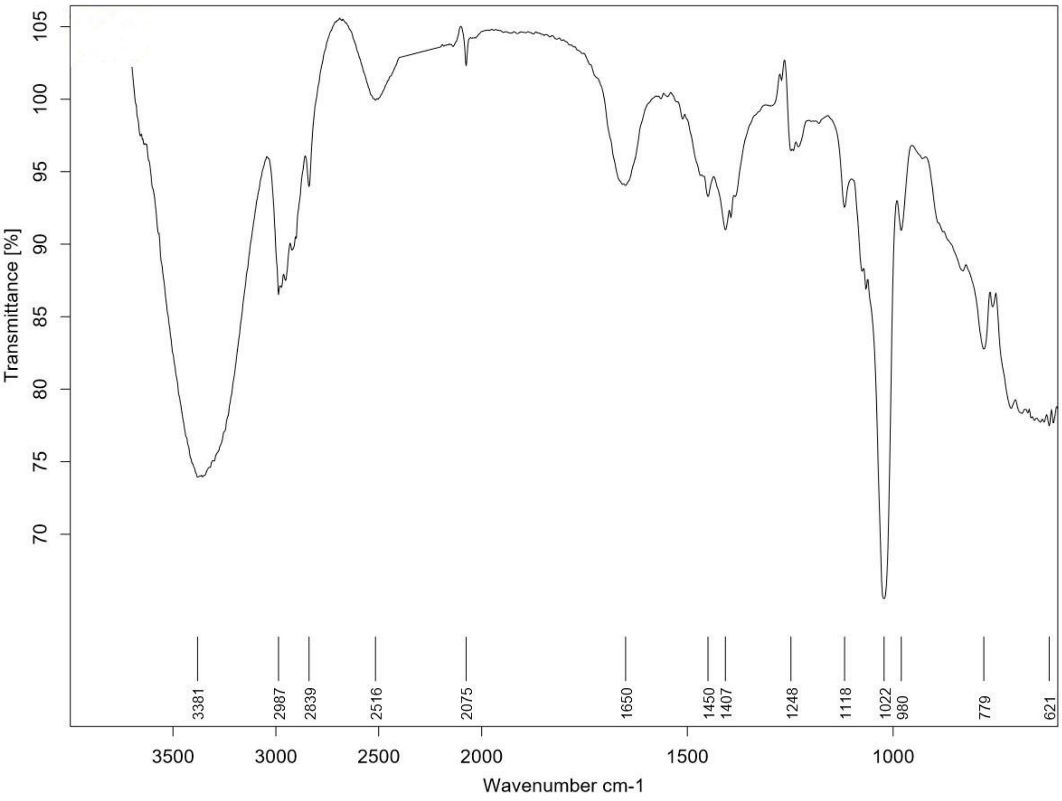

Figure S20. The IR spectrum of compound **3**

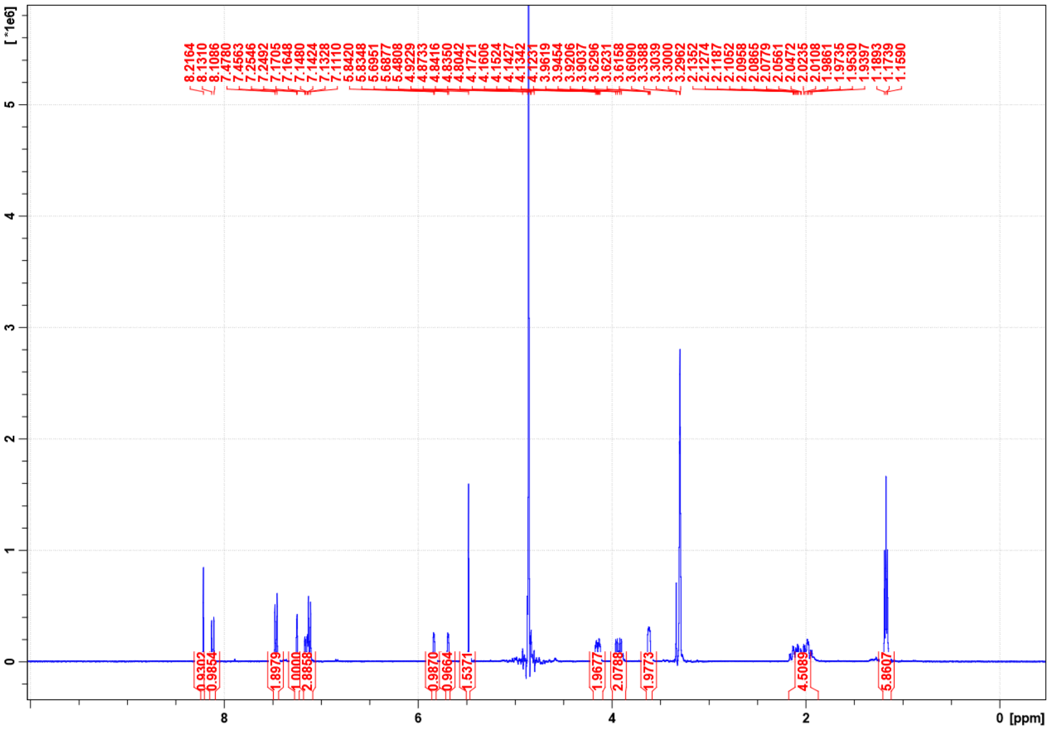

Figure S21. ^{1}H NMR spectrum of compound **3** in CD_3OD (400 MHz)

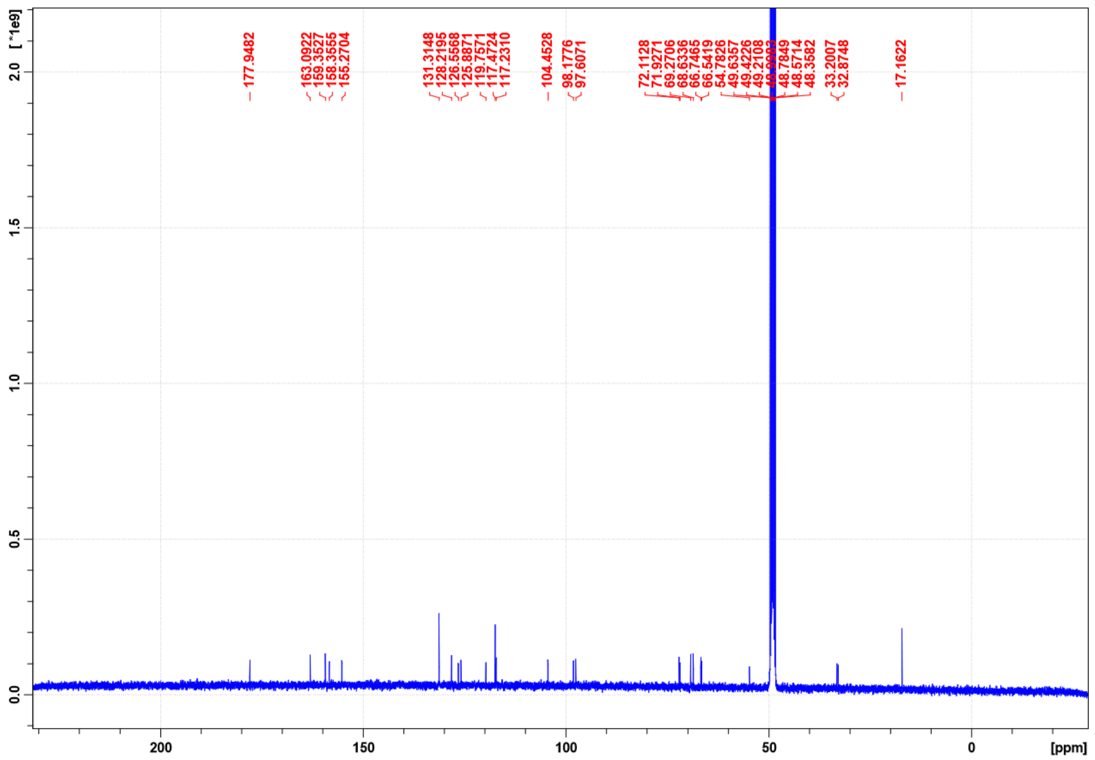

Figure S22. ^{13}C NMR spectrum of compound **3** in CD_3OD (400 MHz)

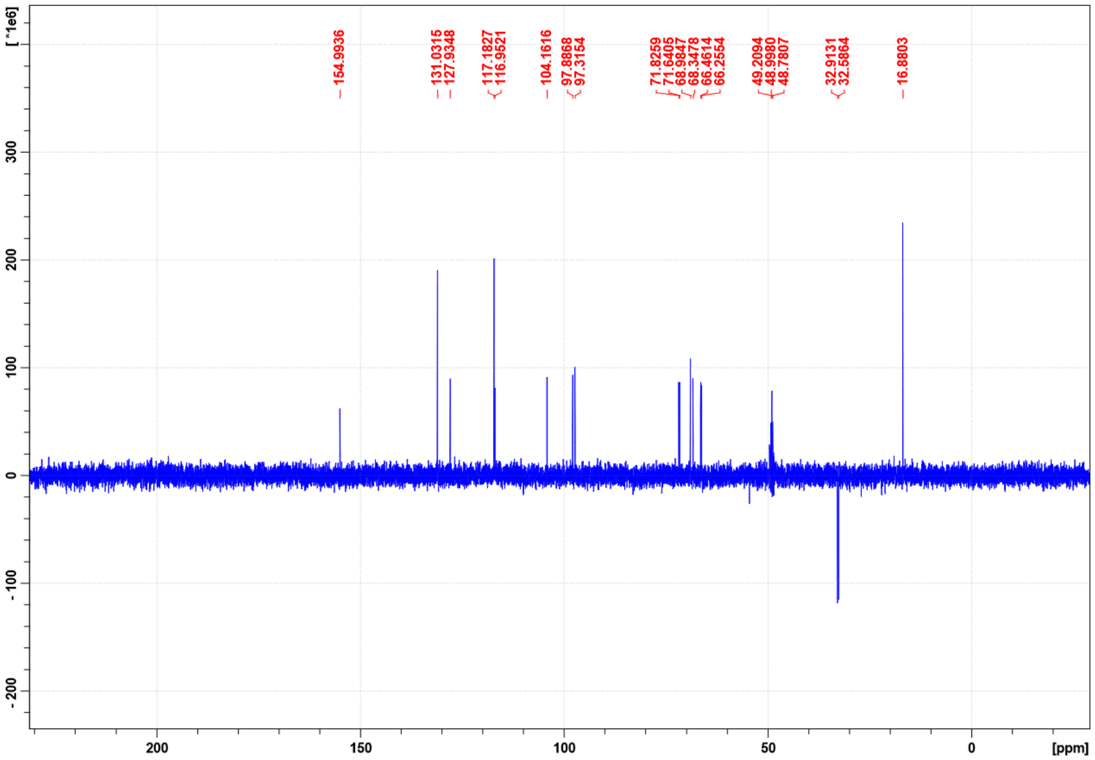

Figure S23. The DEPT spectrum of compound **3** in CD_3OD (400 MHz)

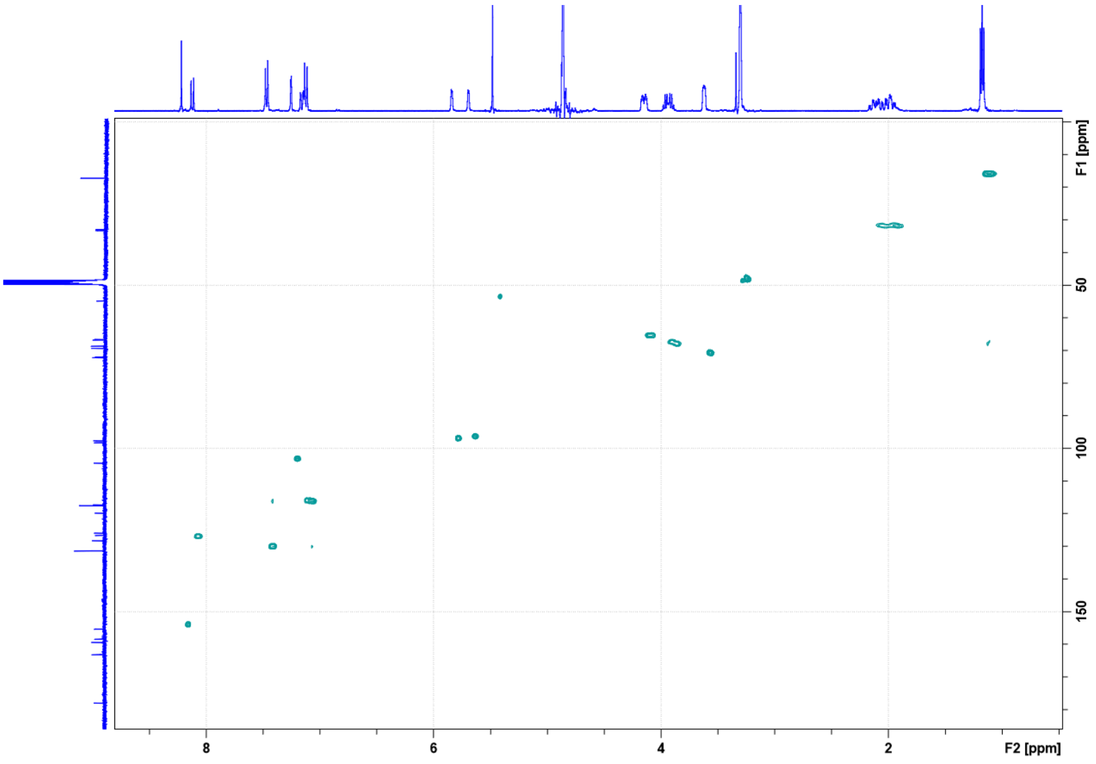

Figure S24. The HSQC spectrum of compound **3** in CD3OD (400 MHz)

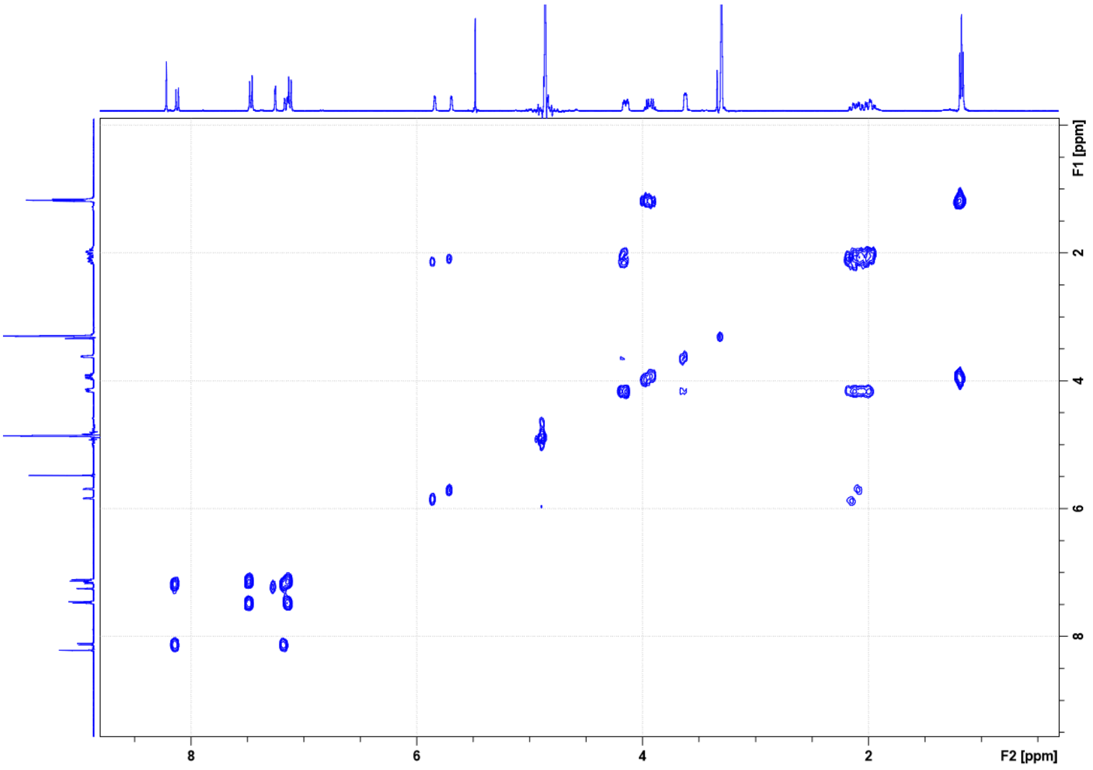

Figure S25. The ^{1}H-^{1}H COSY spectrum of compound **3** in CD3OD (400 MHz)

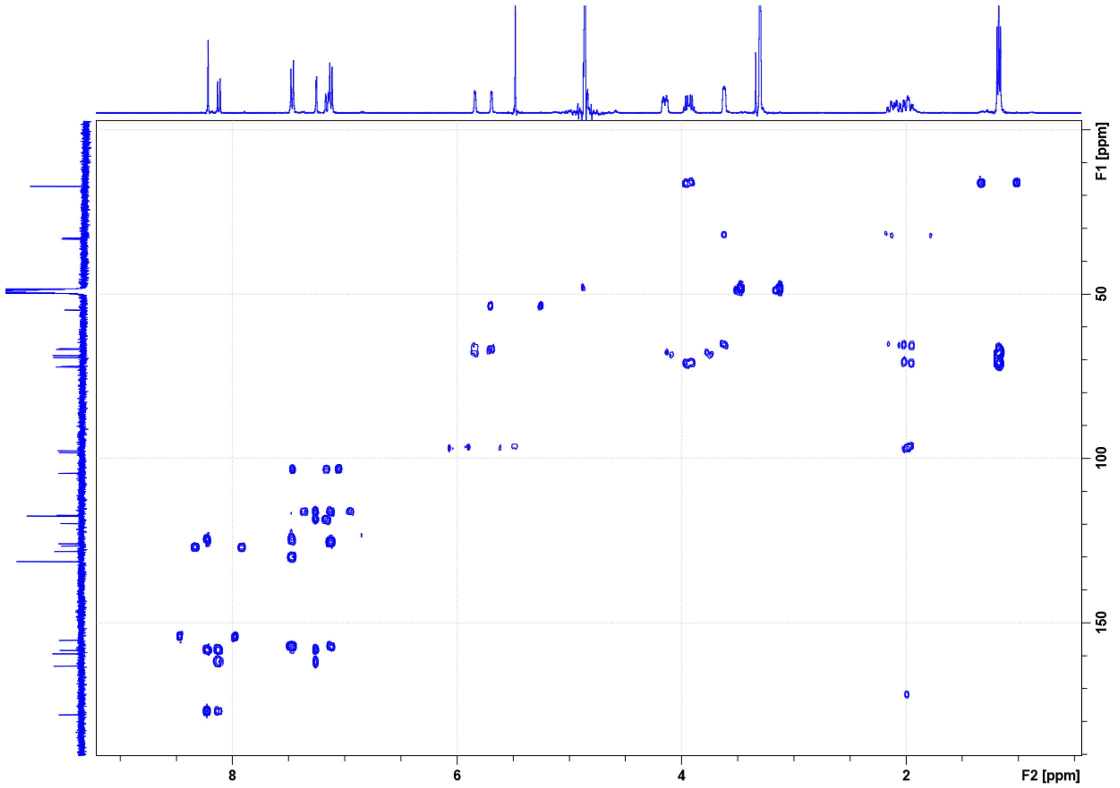

Figure S26. The HMBC spectrum of compound **3** in CD_3OD (400 MHz)

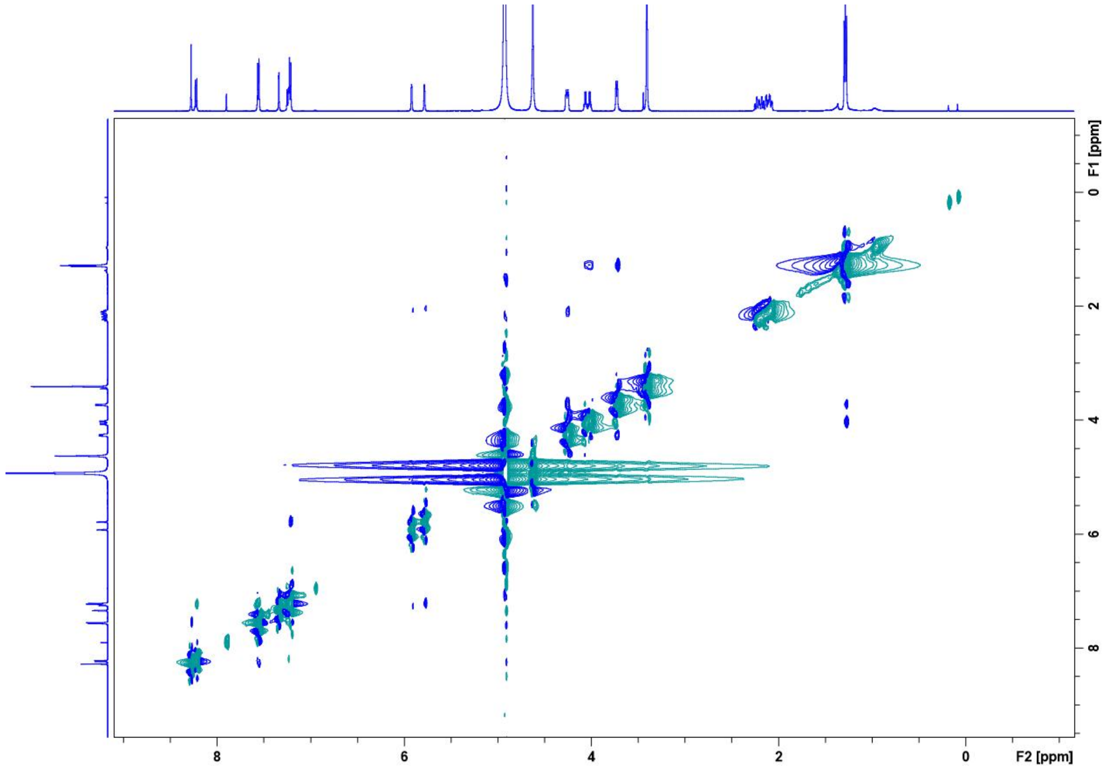

Figure S27. The NOESY spectrum of compound **3** in CD_3OD (600 MHz)

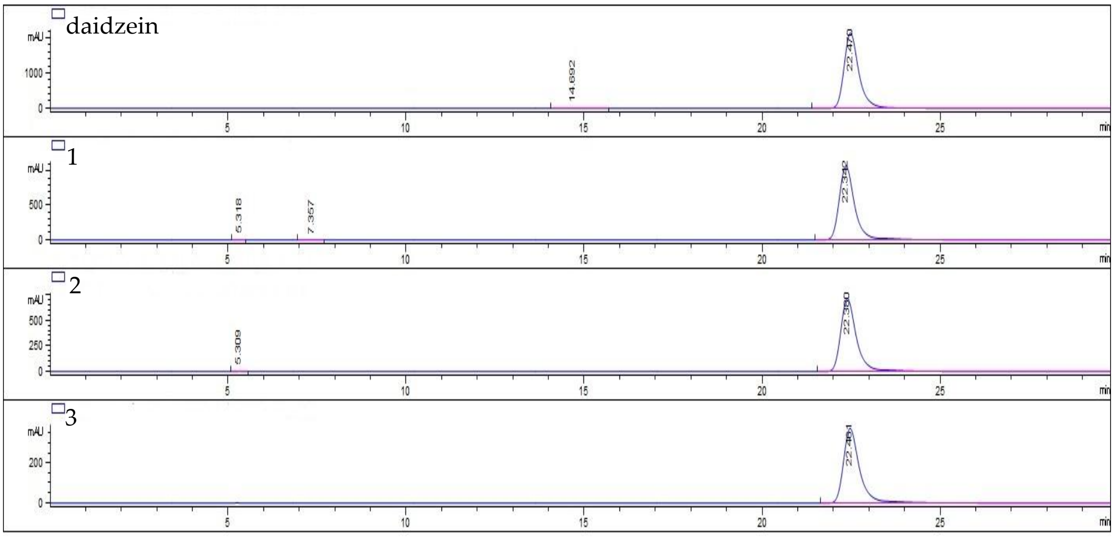

Figure S28. The retention time of daidzein and the aglycone moieties of compounds **1**-**3**

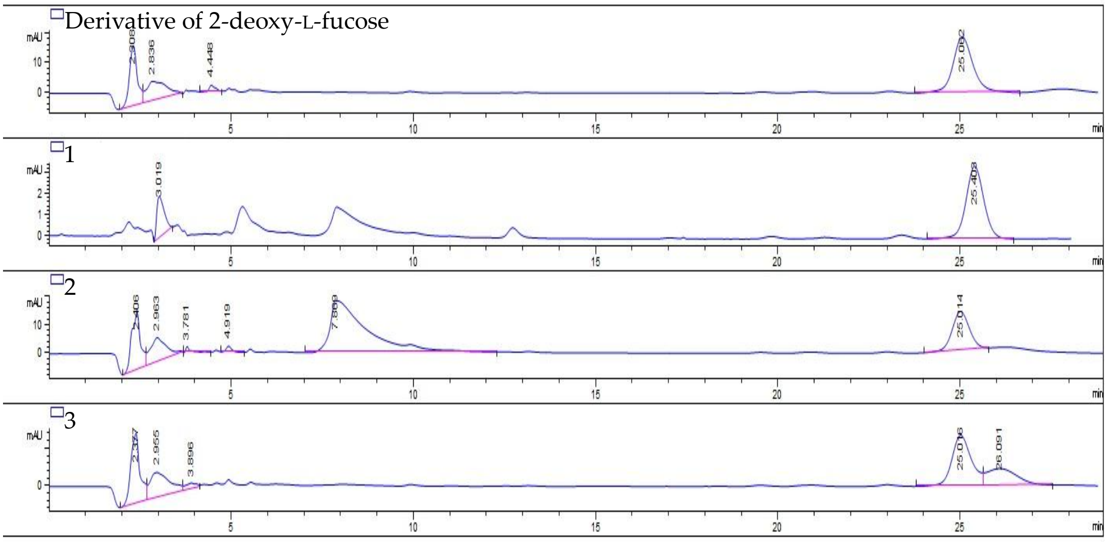

Figure S29. The retention time of the *O*-tolylthiocarbamate derivatives of 2-deoxy-L-fucose and the liberated sugars of compounds **1**-**3**
